# Exercise—A Panacea of Metabolic Dysregulation in Cancer: Physiological and Molecular Insights

**DOI:** 10.3390/ijms22073469

**Published:** 2021-03-27

**Authors:** Steffen H. Raun, Kristian Buch-Larsen, Peter Schwarz, Lykke Sylow

**Affiliations:** 1 Section of Molecular Physiology, Department of Nutrition, Exercise and Sports, Faculty of Science, University of Copenhagen, 2100 Copenhagen, Denmark; raun@nexs.ku.dk; 2 Department of Endocrinology, Rigshospitalet, 2100 Copenhagen, Denmark; kristian.buch-larsen@regionh.dk (K.B.-L.); peter.schwarz@regionh.dk (P.S.); 3 Faculty of Medical and Health Sciences, University of Copenhagen, 2200 Copenhagen, Denmark; 4 Department of Biomedical Sciences, Faculty of Medical and Health Sciences, University of Copenhagen, 2200 Copenhagen, Denmark

**Keywords:** cancer, metabolism, exercise, skeletal muscle, insulin resistance, adipose tissue, cancer cachexia

## Abstract

Metabolic dysfunction is a comorbidity of many types of cancers. Disruption of glucose metabolism is of concern, as it is associated with higher cancer recurrence rates and reduced survival. Current evidence suggests many health benefits from exercise during and after cancer treatment, yet only a limited number of studies have addressed the effect of exercise on cancer-associated disruption of metabolism. In this review, we draw on studies in cells, rodents, and humans to describe the metabolic dysfunctions observed in cancer and the tissues involved. We discuss how the known effects of acute exercise and exercise training observed in healthy subjects could have a positive outcome on mechanisms in people with cancer, namely: insulin resistance, hyperlipidemia, mitochondrial dysfunction, inflammation, and cachexia. Finally, we compile the current limited knowledge of how exercise corrects metabolic control in cancer and identify unanswered questions for future research.

## 1. Cancer Survival Depends on Better Metabolism Management Strategies

The disruption of glucose homeostasis in patients with cancer was already described in the early 20th century [1]. Those observations were later confirmed by studies showing decreased glucose tolerance and reduced insulin responsiveness in patients with various cancers [2,3]. Since then, more cancer site-specific studies have emerged, including insulin resistance and/or glucose intolerance in cancers of the breast [4], pancreas [5], lung [6,7,8], gastro-intestine system [7,8,9], and colon/rectum [8,10]. As cancer survival rates have been increasing, it is important to recognize that cancer survivors often develop obesity and type 2 diabetes (T2D) [11,12], suggesting that metabolic dysregulation persists during the entirety of the cancer diagnosis and even after end of treatment.

While dysregulated metabolism in patients with cancer has been recognized for a long time, it was only recently established that metabolic derangements are associated with a higher risk of cancer incidence and lower survival rates. Obesity, hyperglycemia, hyperinsulinemia, and T2D have all been associated with an increased risk of several cancers [13,14,15,16]. A meta-analysis showed that cancer incidence increased by 20–50% for each 5 kg/m^2^ increase in BMI [17]. Thus, for a 70 kg person with a BMI of 23 (height: ~175 cm), a 15 kg weight gain, resulting in a BMI of 28, would markedly increase the cancer incidence for several of the most common cancers, including those of the breast and colon. Thus, dysregulated metabolism can be seen as an oncogenic condition.

Even more strikingly, once diagnosed with cancer, metabolic dysfunction is associated with poor cancer outcomes, including increased cancer mortality [17,18,19,20,21,22,23,24]. For example, the risk of death from cancer for patients with obesity is 52 percent higher for men and 62 percent higher for women than mortality rates in patients of healthy weight [20,21]. More recent studies also show that diabetes is associated with a 10–30% increased overall risk of cancer death, although the risk markedly varies by cancer site, sex, and treatments [22,25,26]. In fact, cancer has now overtaken vascular disease as leading cause of excess death associated with diabetes [27], clearly illustrating the need for better metabolic management in cancer. Furthermore, metabolic dysregulation persists in patients who survive their cancer [11,12]. As metabolic dysfunction in cancer survivors increases recurrence rates [24,28], better metabolic management is also warranted in cancer survivorship. Exercise is a safe treatment option that addresses several facets of the underlying metabolic abnormalities in cancer as will be discussed below.

**Figure 1:**
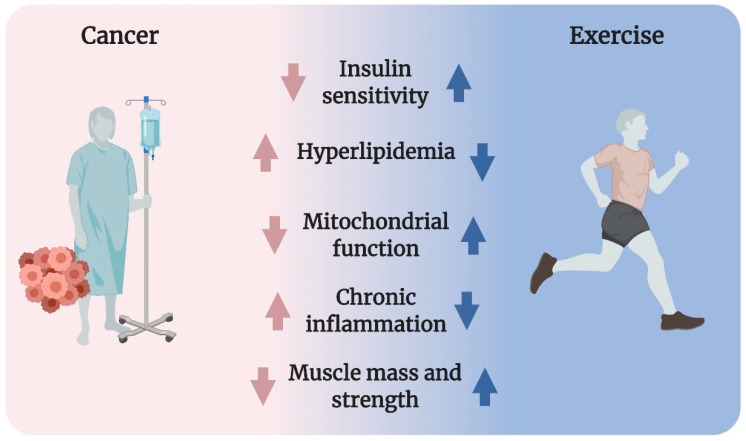
Cancer is often associated with reduced insulin sensitivity, hyperlipidemia, impaired mitochondrial function, chronic inflammation, as well as lowered muscle mass and strength (cancer cachexia). Exercise powerfully improves all of these conditions in healthy humans, suggesting that exercise could be a strategy to counter metabolic derangements in cancer.

The metabolic dysfunctions in cancer (described in detail in Section 3.1, Section 3.2, Section 3.3, Section 3.4 and Section 3.5) include reduced insulin sensitivity, hyperlipidemia, impaired mitochondrial function, chronic inflammation, and cancer cachexia (Figure 1). To date, there have been no satisfactory answers to the fundamental question of what initiates the metabolic dysfunctions observed in patients with cancer. Possibilities include tumor-derived factors, cancer treatment side effects, and/or the fact that metabolic dysfunction is associated with increased risk of cancer.

Better metabolic management strategies are needed for people diagnosed with cancer because it may improve overall survival. Therefore, the aims of this review are to: (i) compile the current data on the processes that are dysfunctional in cancer, (ii) to describe the known mechanisms by which exercise enhances metabolic regulation in healthy people, and (iii) to extrapolate such mechanisms to discuss how exercise could be a panacea for metabolic dysregulation in cancer.

## 2. Exercise as a Panacea in Metabolic Dysregulation in Cancer

A multiplicity of health benefits arises from exercise during and after cancer treatment [29,30] likely due to improved metabolic regulation. Yet, the effect of exercise on metabolism in cancer is surprisingly understudied. Regarding metabolic control, skeletal muscle is of special interest because it is a major site for insulin- and meal-dependent glucose disposal from the blood in humans [31,32]. Exercise potently improves insulin sensitivity, hyperlipidemia, mitochondrial function, inflammation, muscle mass, and strength (Figure 1) [33,34]. Because of those potent effects, which are superior to any known drug, exercise is the standard of care in several conditions associated with dysregulated metabolism, including T2D, cardiovascular diseases, and obesity [33].

## 3. Potential Mechanism Underlying Cancer-Associated Metabolic Dysregulation

**Figure 2:**
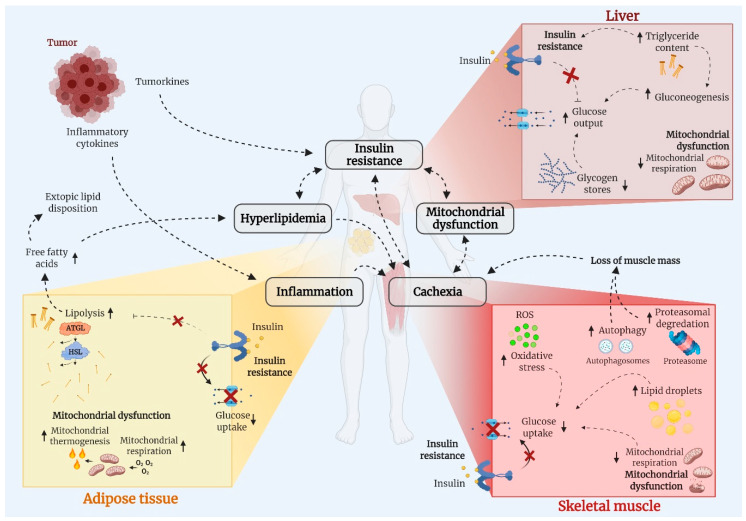
Cancer is associated with adverse changes of metabolic important tissues, including skeletal muscle (myocytes), adipose tissue (adipocytes), and the liver (hepatocytes). Those changes include insulin resistance, hyperlipidemia, mitochondria dysfunction, inflammation, and muscle mass loss (cancer cachexia) and there is significant tissue crosstalk. Abbreviations: ATGL; adipose triglyceride lipase, HSL; hormone sensitive lipase, ROS; reactive oxygen species.

Whole-body metabolic homeostasis is tightly balanced. In response to a meal, insulin is secreted from the pancreatic β-cells and reduces circulating glucose and fatty acids by promoting uptake into skeletal muscle [35] and adipose tissue while inhibiting hepatic glucose output and adipose tissue lipolysis [36]. These mechanisms contribute to restoring homeostasis after a meal and impairments in these processes will therefore produce metabolic dysregulation, such as hyperglycemia, hyperlipidemia, and hyperinsulinemia. Cancer cells rely heavily on glucose metabolism to sustain the continuous proliferation and cell growth described as the Warburg Effect [37], which might result in divergence of glucose from other tissues to the cancer cells as suggested in for example leukemia [38]. Yet, the cause of metabolic derangements in cancer has not been identified. Obesity is a risk factor of several cancers [17] and, therefore, obesity *per se* might contribute to metabolic derangements often observed in patients with cancer. Nevertheless, molecular and metabolic processes within skeletal muscle, adipose tissue, and the liver are markedly influenced by cancer, as illustrated in Figure 2. Emerging evidence suggests that cancer-associated metabolic dysfunction is due to insulin resistance, hyperlipidemia, mitochondrial dysfunction, inflammation, and/or muscle wasting (cachexia). These processes will now be discussed.

### 3.1. Insulin Resistance 

Insulin resistance is a condition of reduced responsiveness to insulin. Thus, more insulin is needed to obtain a given physiological outcome, such as lowering of blood glucose. In insulin-resistant conditions, the β-cells of the pancreas compensate by escalating insulin secretion to produce hyperinsulinemia. As long as compensatory hyperinsulinemia is maintained, normoglycemia can be upheld. Yet, hyperinsulinemia can be detrimental in cancer, because insulin and IGF-1 promote cancer cell growth [39]. In obesity and T2D, skeletal muscle insulin resistance is considered a primary cause of dysregulated metabolism [31,35], yet, the contribution from skeletal muscle insulin resistance to the metabolic derangements associated with cancer has not been directly determined. Using the hyperinsulinemic-euglycemic clamp, the gold standard for measuring insulin sensitivity, whole-body insulin resistance has been established in pancreatic [5], lung [6,7,8], gastrointestinal system [7,8,9], and colorectal [8,10] cancers; although none of those studies determined tissue-specific glucose uptake. Recent data from preclinical models of cancer have shed some light on this. Using isotopic tracers, we recently showed that insulin-stimulated glucose uptake into skeletal muscle and adipose tissue was markedly reduced in Lewis Lung Carcinoma (LLC) bearing mice [40]. Preclinical rodent models of cancer also recapitulated the reduced blood-glucose-lowering effect of insulin, finding insulin resistance [40,41,42] and glucose intolerance [40,43] compared to controls.

The cause of reduced skeletal muscle glucose uptake in cancer is incompletely defined but was observed with a concurrent decrease in insulin-stimulated microvascular perfusion of muscle in tumor-bearing mice, which is similar to what has been observed in insulin-resistant obese subjects [44]. In healthy skeletal muscle, insulin stimulates the translocation of the glucose transporter 4 (GLUT4) to the sarcolemma and transverse tubules of the muscle membrane to facilitate glucose uptake (reviewed in [35]). This process is, in part, regulated by the intracellular activation of Rac1, Akt, and TBC1D4 [45,46,47,48]. However, despite impairments in insulin-stimulated glucose uptake in tumor-bearing mice, signaling via Akt and TBC1D4 was, surprisingly, elevated [40]. Thus, the mechanisms by which cancer causes insulin resistance in vivo seem to be different from those causing insulin resistance in obesity and T2D, where muscle Akt and TBC1D4 signaling is either unaffected [49,50] or reduced [51,52].

In addition to skeletal muscle and adipose tissue insulin resistance, data also suggest hepatic insulin resistance in cancer, although the studies are few. Increased basal hepatic glucose production and increased hepatic gluconeogenesis have been reported in patients with lung cancer [53,54]. Similarly, we observed 45% elevated basal hepatic glucose production in tumor-bearing female mice [40] and, while we found a preserved inhibitory effect of insulin on hepatic glucose output [40], Lang et al. reported impaired insulin suppression of hepatic glucose production in tumor-bearing rats [42]. Increased hepatic glucose output may be due to increased gluconeogenesis because increased metabolic-intermediates and increased gene-expression of markers of gluconeogenesis have been observed in the liver of tumor-bearing animals [55]. A recent proton nuclear magnetic resonance (1H-NMR) metabolomics study of cachexia in the mouse colon carcinoma 26 (C26) model revealed reduced glucose metabolism and increased lipid metabolism in the liver [56]. Whether hepatic metabolic changes in cancer is induced by insulin resistance or is a contributing factor to insulin resistance remain unidentified.

### 3.2. Hyperlipidemia

Hyperlipidemia is an umbrella term that refers to conditions associated with levels of lipid (free fatty acids (FFA), cholesterol, and triglycerides) circulating in the blood that is persistently elevated above baseline. In cancer, hyperlipidemia has been reported in human cancers [57,58,59,60] and pre-clinical cancer models [40,43,61,62,63,64,65]. Cancer-associated hyperlipidemia is likely caused by accelerated lipolysis in adipose tissue due to the upregulation of the key lipolytic enzymes, AGTL and HSL in both humans [61,66] and rodent pre-clinical models [61].

Hyperlipidemia in cancer could also be implicated in cancer cachexia in addition to the development of insulin resistance. Regarding the latter, high levels of circulating FFA have been linked to muscle and liver insulin resistance in obesity and T2D [67,68,69]. Thus, hyperlipidemia could contribute to insulin resistance in cancer. Accordingly, fatty acid inhibition by etomoxir or lipolysis inhibition by nicotinic acid largely restored insulin sensitivity and glucose intolerance in tumor-bearing mice [40]. The relationship between hyperlipidemia and insulin resistance is bi-directional as a key physiological function of insulin is to restrain lipolysis and to promote fat storage in adipose tissue in the postprandial state [70], making it likely that insulin resistance contributes to hyperlipidemia in cancer.

Excessive fatty acid turnover is in particular associated with cancers causing cachexia. In patients with cancer, total lipase, ATGL, and HSL activities were significantly higher in visceral white adipose tissue compared with individuals without cancer [66]. When stratifying the results between patients with and without cachexia, lipase activities were significantly higher in patients with cancer cachexia compared with patients without cachexia [66]. Accordingly, high levels of circulating sphingolipids was observed in murine and human cancer cachexia [71]. Hyperlipidemia also seems to reach the muscle, as the number of intramyocellular lipid droplets was increased in patients with cancer and positively correlates with involuntary weight-loss [72]. Altered lipid profile might be a causative mechanism of cancer cachexia (further discussed in Section 3.5), because blockade of fatty acid oxidation or suppression of lipolysis in adipose tissue prevents cachexia in tumor-bearing mice [61,73].

Under pathological conditions of excessive adipose tissue lipolysis, the liver acts as a major sink for adipose tissue–derived FFA. Accordingly, hepatic lipids and triglycerides were elevated 3–5 fold in colon (C26) tumor-bearing mice, an effect that was not due to reduced food intake [56,74]. The hepatic steatosis could be explained by a reduction in carnitine and carnitine biosynthesis, decreased VLDL excretion, reduction in lipogenic genes, and an upregulation fatty acid transporters in the liver [56,74]. Within the hepatocyte, non-esterified FAs are oxidized to mitochondrial acetyl CoA resulting in elevated gluconeogenic flux. This, together with net glycogenolysis, facilitates hepatic glucose production [75] whereby excessive hepatic lipid accumulation could contribute to the elevated hepatic glucose output in cancer described in Section 3.1. Thus, there is potentially a contributing role of tissue crosstalk between adipose lipolysis and hepatic gluconeogenesis that contributes to metabolic dysfunctions in cancer.

### 3.3. Mitochondrial Dysfunction

Mitochondrial dysfunction might contribute to muscular insulin resistance in the presence of ectopic accumulation of fat [76]. Mitochondria generate most of the cell’s supply of adenosine triphosphate (ATP), used as a source of chemical energy, but mitochondria are also important signaling organelles that integrate metabolic signals to provide important cues to muscle mass maintenance [77]. Recent proteomic analyses of skeletal muscle from patients with gastrointestinal [78] or breast [79] cancer found reduced expression of mitochondrial proteins and proteins involved in oxidative phosphorylation. Furthermore, skeletal muscle mitochondrial dysfunction has been confirmed in multiple rodent preclinical models of cancer [80,81,82,83,84,85,86,87,88,89] and drosophila [90]. Additionally, swollen mitochondria and increased mitochondrial area have been observed in skeletal muscle from patients with gastric or colon cancer [91]. But this is not a consistent finding since a decreased mitochondrial area has been observed in muscle from patients with breast cancer [92], although function was not tested.

Dysfunctional mitochondria can lead to the production of reactive oxygen species (ROS) and oxidative stress, as seen in metabolic diseases such as T2D [93]. Together with increased lipid flux to muscle, mitochondrial dysfunction and/or elevated production of ROS likely contribute to the diminished glucose uptake response of insulin-resistant muscle [94]. Similarly, cachectic rodent muscle develops oxidative stress [95,96,97], which may further compromise cellular functions.

In addition to muscle, dysregulated mitochondria are present in the liver of preclinical cancer models [86,98,99] and in hepatocytes incubated with various cancer-conditioned media [100]. Interestingly, the opposite is true for adipose tissue, which displays increased mitochondrial respiration [86,101] and mitochondrial proteins in rodent cancer cachexia. Furthermore, a switch from white to brown fat has been proposed to increase energy expenditure in cancer-associated cachexia [101] but the current human data implies a limited role for fat browning in cancer [102,103].

### 3.4. Chronic Inflammation

Chronic inflammation is well described in cancer [104,105]. Whether caused by tumor-extrinsic and/or -intrinsic factors, chronically elevated circulating levels of IL-6, IL-1, and TNF-α would be expected to skew immune cells residing within skeletal muscle towards proinflammatory phenotypes [76,106]. By secreting proinflammatory molecules, immune cells may induce myocyte inflammation, adversely regulate myocyte metabolism, and contribute to insulin resistance via paracrine effects. Increased influx of fatty acids and inflammatory molecules from other tissues, particularly visceral adipose tissue, can also induce muscle inflammation and negatively regulate myocyte metabolism, leading to insulin resistance. Yet, inflammation did not seem to be a main driver of insulin resistance in a recent study [40]. Plasma IL-6 and TNF-α were similarly increased in mice with small and large tumors, while only the mice with large tumors displayed reduced insulin-stimulated glucose uptake in muscle and adipose tissue [40]. This agrees with data showing that lipid-induced insulin resistance is not always accompanied by inflammation in humans [68].

In contrast to insulin resistance, cancer cachexia may be driven by inflammation as and IL-6, IL-1, and TNF-α *per se* can cause muscle catabolism [107,108]. In preclinical models, antibody administration against IL-6 [109,110] or IL-6 knockout [111] prevents cancer cachexia, although anti-inflammatory treatments have shown less positive outcomes in human trials (reviewed in [112]). Thus, more studies are warranted to determine if inflammation contributes to dysregulated metabolism in cancer.

### 3.5. Cancer-Associated Cachexia

Cancer-associated cachexia, which is the involuntary progressive loss of both muscle and fat mass [113], affects 50–80% of all patients with cancer. Cachexia is associated with specific tumor types such as pancreatic, oesophageal, gastric, lung, and liver, and patients with these malignancies have the highest degree of weight loss. Cachexia is defined as loss of >5% of body mass within six months [113], which is associated with poor metabolic control, reduced quality-of-life, treatment intolerance, and increased mortality [8,10,114,115,116].

Cachexia is associated with altered protein metabolism, including decreased basal protein synthesis and accelerated skeletal muscle protein degradation through mechanisms reviewed in [117,118,119,120,121]. In cachectic skeletal muscle, basal protein synthesis is decreased in both humans [122] and rodent [110,123,124,125] models. Molecularly, this could be due to decreased mTORC1-S6K signaling, which has been observed in skeletal muscle from patients with lung cancer cachexia [126].

In addition, cachexia is associated with accelerated protein degradation indicated by increased skeletal muscle expression of the E3 ligases, atrogin-1 and MuRF-1, and ubiquitin involved in proteasomal degradation and increased autophagy in humans [91,127,128,129]. What is perplexing is that not all cancer patients with a similar tumor burden develop cachexia. 

Because skeletal muscle is a major site for glucose disposal, muscle mass loss in cachexia could contribute to dysregulated metabolism in cancer. While this remains to be proven, age-associated unwanted muscle mass loss, sarcopenia, is associated with increased HOMA-IR (a measure of insulin resistance) and hyperglycemia [130], suggesting that muscle mass loss *per se* could lead to a dysregulation of glucose metabolism. “Anabolic resistance” has been described in older subjects with sarcopenia, which manifests as a lack of increase in muscle protein synthesis in response a meal, which has been partially ascribed to insulin resistance [131,132,133]. Whether similar mechanisms are in play in cancer cachexia have not been established but is imperative as anabolic resistance in cancer could contribute to cachexia. The relationship between cancer cachexia and insulin resistance is bi-directional as shown in Figure 2. Thus, insulin resistance by itself has been suggested to be a causative factor for cancer cachexia in drosophila flies [90,134]. Once insulin resistance was genetically corrected in these models, cancer cachexia was also abolished. Accordingly, cancer-associated insulin resistance occurs before the onset of muscle mass loss in rodent cancer models [40,41], and targeting insulin resistance might provide new treatment options for cancer cachexia.

## 4. Exercise Is the Most Powerful Means to Improve Metabolic Regulation

**Figure 3:**
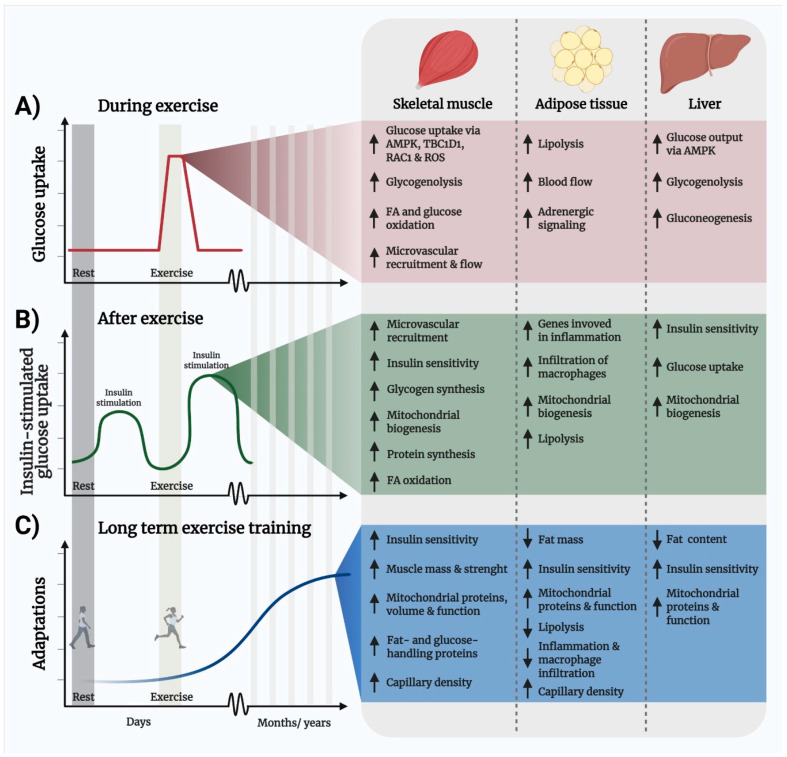
Illustration of the temporal metabolic benefits of exercise and molecular mechanisms. (**A**) One exercise bout elicits an acute and transient increase in muscle glucose uptake that is independent of insulin and persists in insulin-resistant subjects. (**B**) Insulin sensitivity (illustrated here as insulin-stimulated glucose uptake) is transiently enhanced for up to 48 h after the last exercise bout. (**C**) Repeated exercise training leads to longer-term adaptations, including increased expression of fat- and glucose-handling proteins and increased capillarization that improves insulin sensitivity and elevates muscle fat- and glucose-handling capacity. Abbreviations: AMP-activated protein kinase, FA; fatty acid, RAC1; Ras-related C3 botulinum toxin substrate 1, ROS, reactive oxygen species, TBC1D1; TBC1 Domain Family Member 1.

Exercise is a safe and effective intervention to improve metabolic health. Exercise is therefore part of the standard of care for several lifestyle-related conditions such as obesity, cardiovascular disease, and T2D [34,135,136]. Exercise improves metabolic regulation via both acute events as well as chronic adaptations as illustrated in Figure 3. The acute beneficial metabolic effects of exercise can be divided into two phases: (i) the physiological events that occur during the exercise bout, which includes an up to 50-fold increase in glucose uptake by the working muscles (Figure 3A), and (ii), the events occurring in the hours to days that follow a single bout of exercise, including a transient increase in insulin sensitivity (Figure 3B). The longer-term benefits from weeks and months of repeated bouts of exercise, termed exercise training (ET), include increased insulin sensitivity, improved mitochondrial volume and function, and increased muscle mass and strength from resistance exercise (Figure 3C). Thus, because exercise improves insulin resistance, hyperlipidemia, mitochondrial function, inflammation, and promotes muscle mass growth, exercise might correct the majority of metabolic dysfunctions in cancer. Below we will describe the mechanisms of this novel treatment paradigm and place them in “the context of cancer” by presenting the current evidence that exercise can be used to treat the dysregulated metabolism in cancer.

### 4.1. Exercise Improves Insulin Sensitivity and Glucose Disposal

During an exercise bout (Figure 3A), the working muscles markedly increase the uptake of glucose and fatty acids [137]. Exercise-stimulated glucose uptake is independent of insulin [138,139], producing a powerful alternative mechanism to clear glucose from the blood in insulin-resistant skeletal muscle. The mechanisms by which exercise stimulates glucose uptake in skeletal muscle has been extensively reviewed in [140]. In short, exercise simultaneously increases nutrient and oxygen delivery to muscle and prompts a mechanical stress and a metabolic stress in muscle that activate separate but interacting signaling pathways including Rac1, the ROS-producing NADPH oxidase (NOX) 2 complex, and AMP-activated Protein Kinase (AMPK) [141,142,143,144,145,146,147,148,149]. After each exercise bout (Figure 3B), muscle insulin sensitivity is transiently increased [150,151,152,153,154,155] for up to 48 h in humans [156]. The mechanisms by which insulin sensitivity is increased following exercise are not completely understood but they include elevated microvascular recruitment due to increased vasodilatory effect of insulin [157] as well as AMPK-dependent intracellular signaling in muscle [153,158,159,160]. Conversely, physical inactivity, which is often a consequence of cancer, leads to decreased insulin action [161,162,163]. Thus, an acute bout of exercise instantly helps to remove glucose and lipids from the circulation and can mitigate insulin resistance by enhancing insulin-stimulated glucose uptake for up to two days after exercise cessation. As an example, subjects with T2D showed improved glycemic control and lower plasma insulin concentrations 24 h following an acute exercise session [164].

While a single bout of exercise causes a transient increase in insulin independent glucose uptake and subsequent gain in insulin sensitivity, repeated ET leads to a longer-lasting elevation in insulin sensitivity and substrate handling capacity [165,166,167] (Figure 3C). Underlying adaptations of the exercise-trained muscle include enhanced muscle protein expression of glucose-handling proteins (GLUT4, hexokinase II, Glycogen synthase, and pyruvate dehydrogenase), fat-handling proteins (CD36, FATP1/4, ATGL, and CPT-1), increased mitochondrial mass and function, as well as increased muscle capillarization (recently reviewed in [136]).

Taken together, exercise improves insulin sensitivity and glucose disposal, which would be expected to greatly benefit patients with cancer-associated insulin resistance and mitigate the oncogenic condition of hyperinsulinemia [39].

#### Exercise and Insulin Sensitivity in the Context of Cancer

No study has, to our knowledge, determined the effect of an acute exercise bout on insulin sensitivity, nor the efficacy of exercise to stimulate glucose uptake in muscle in patients with cancer. Yet, emerging evidence suggests that longer term ET benefits metabolic regulation in subjects with cancer. For example in prostate cancer, insulin resistance and changes in body composition are side effects of androgen deprivation therapy, and aerobic training has been shown to increase peripheral tissue insulin sensitivity and elevate muscle protein content of GLUT4 [168].

Studies in cancer survivors, have reported indirect measures of insulin resistance, such as glucose tolerance or blood glucose and hormone measures. For example, a recent study found that prescribing 150 min/week of aerobic exercise for 6 months to colorectal cancer survivors lowered serum insulin concentrations [169]. This is supported by data from a 12-week home-exercise intervention consisting of aerobic and bodyweight strength exercises that decreased plasma insulin-concentrations and improved the HOMA-IR index in colorectal cancer survivors [170]. In a recent randomized controlled trial, the effects of a 16-week combined aerobic and resistance exercise intervention on metabolic syndrome, sarcopenic obesity, and serum biomarkers was assessed in sedentary, overweight, or obese survivors of breast cancer [171]. In that study, metabolic regulation and circulating biomarkers such as insulin, IGF-1, leptin, and adiponectin, were significantly improved post-intervention compared with usual care. Yet, another study reported that 12-week aerobic ET had limited overall benefits on insulin concentrations following glucose ingestion in breast cancer survivors [172]. In female patients diagnosed with various cancers, 10-weeks of combined aerobic and resistance ET led to increased expression of skeletal muscle GLUT4 protein [173], suggesting that ET causes insulin-sensitizing molecular adaptations in skeletal muscle of cancer patients [173].

In mice, the cachexic *Apc^min/+^* mouse cancer model develops glucose intolerance concurrently with the development of cachexia [43]. Forced treadmill running ET did not restore glucose tolerance in this model [174]. Yet, ET also failed to improve glucose tolerance in the non-tumor control mice [174], which is in agreement with other reports that forced ET does not produce metabolic benefits in mice, likely due to stress [175]. In contrast, voluntary wheel running ET improves glucose tolerance in mice [176,177,178,179,180] and should therefore be the preferred model of choice to determine whether ET improves metabolic health and insulin sensitivity in preclinical models of cancer. Taken together, longer-term ET seems to benefit some metabolic parameters, such as lowering circulating insulin levels and elevating GLUT4 protein content in cancer patients or survivors, but it remains to be determined whether exercise elicits acute benefits for patients with cancer.

### 4.2. Hyperlipidemia Can Be Managed by Exercise

During acute prolonged exercise, plasma FFA levels increase primarily due to increased adipose tissue blood flow and lipolysis [181,182], partly via adrenergic signaling [183,184], which is also elevated in the recovery period after exercise cessation [182,185] to support the increased fat oxidation by muscle in response to exercise (reviewed in [186]). While acute exercise causes a transient increase in plasma FFA levels, long-term ET enhances insulin sensitivity and increases vascularization of adipose tissue [187], that in turn potentiates insulin’s inhibitory effect on lipolysis in response to for example a meal [188]. In addition, the effect of exercise on postprandial lipemia (excess of lipids in the blood) has been studied for many year [189]. Here, both acute exercise [190,191] and ET [192,193,194,195] reduce postprandial lipemia in humans. The ET effect is likely, in part, due to an upregulation of fat-handling proteins in both skeletal muscle and adipose tissue including CD36, FATP1/4, ATGL and CPT-1 [196,197,198,199,200], thereby improving fat metabolism on a whole-body level.

In agreement with the fat-liver cross talk described in Section 3.2, emerging evidence clearly demonstrates that ET, can effectively reduce intrahepatic lipids in for example adults with T2D [201] and adolescent boys with obesity [202] with minimal changes in total body mass. Exercise uniquely prepares the liver for excess delivery of FFAs by leading to upregulation of hepatic fatty acid oxidation, improving mitochondrial respiration, and by increasing other associated mitochondrial outcomes such as citrate synthase activity, β-HAD activity, cytochrome c content, which has been reviewed in [203]. These increases in markers of hepatic oxidative capacity are retained chronically in the liver and could be beneficial for handling of the excessive circulating FFA and elevated hepatic TG content often observed in cancer.

#### Exercise and Hyperlipidemia in the Context of Cancer

In prostate cancer patients, a two-year home-based ET [204] or a 12-weeks endurance ET [168] intervention decrease body fat mass [168,204] and plasma triglycerides [204]. In a preclinical hyperlipidemic rodent cancer model, an aerobic ET intervention lowered the elevated levels of plasma triacylglycerol and LDL and increased HDL [174]. Although not tested in the context of cancer, increasing adipose tissue insulin sensitivity by exercise could lower cancer-associated accelerated lipolysis to protect against cancer cachexia, as tissue loss can be prevented by lipolysis inhibition in preclinical rodent models [61,73]. Lastly, cancer-associated glucose intolerance and insulin intolerance can be ameliorated by inhibition of fatty acid oxidation and lipolysis inhibition in mice [40], providing a rationale for managing hyperlipidemia with exercise in cancer. Clinical trials are needed to determine this in humans.

### 4.3. Skeletal Muscle Mitochondrial Volume and Function Is Upregulated by Exercise

First shown in 1967 by Prof. Holloszy [205], the mitochondrion is a highly exercise-sensitive organelle in skeletal muscle, where both mitochondrial volume, proteins, and function are potently upregulated by ET [206]. In addition to changes in the working muscles, ET also increases mitochondrial biogenesis in white adipose tissue in human [207,208,209,210] and in rodents [211,212,213] (although this is not a consistent finding in humans [214,215]). Increased mitochondrial biogenesis and respiration in liver after ET has also been observed in rats [216,217]. At a molecular level, ET leads to a bulk of signaling events, that include the activation of peroxisome proliferator-activated receptor γ co-activator-1α (PGC-1α), which induces mitochondrial biogenesis, as well as the activation of a myriad of other signaling pathways extensively reviewed in [218,219] that conjointly increases mitochondrial volume, quality, and function [218,219]. Considering the relationship between mitochondrial dysfunction and insulin resistance proposed in T2D [220], and the mitochondrial dysfunctions described in cancer (please see Section 3.3), ET-induced mitochondrial adaptation may improve metabolic regulation in cancer.

#### Exercise-Induced Mitochondrial Improvements in the Context of Cancer

Mitochondria are a putative target in relation to both the metabolic dysfunctions and the loss of muscle mass in cancer. Multiple studies have shown that ET in tumor-bearing animals leads to increased mitochondrial proteins and enzyme activity in skeletal muscle [88,89,97,174,221,222,223,224]. Furthermore, ET can reduce cancer-associated oxidative stress in preclinical models [95,96,97]. Whether enhanced mitochondrial function improves metabolic regulation in cancer is uncertain. For example, in the previously described study in *Apc^min/+^* mice, ET had no effect on glucose intolerance in a murine cancer model despite increased mitochondrial proteins in skeletal muscle [174]. In clinical studies, breast cancer patients undergoing chemotherapy had an increase in citrate synthase activity and protein expression of complexes in the electron transport chain in skeletal muscle, after 16-weeks of aerobic ET [225]. The same study also showed that, in relation to mitochondrial proteins, aerobic ET was superior to resistance ET [225], which is in accordance with studies in healthy humans. However, another study found no effect of ET on the expression of several mitochondrial proteins in skeletal muscle during chemotherapy for various cancer types [173]. This suggests that the cancer itself and/or the treatment could reduce ET-induced mitochondrial adaptations. Taken together, it is possible that ET may improve mitochondrial function in people with cancer but this benefit must be explored in detail.

### 4.4. Exercise Lowers Chronic Inflammation

Both aerobic- and resistance ET interventions decrease plasma markers of chronic inflammation such as C-reactive protein, IL-6, and interferon-γ in older-aged people with or without T2D [226,227]. In mouse adipose tissue, ET leads to decreased expression of IL-1β [228], IL-12 [228], TNF-α [229], and MCP-1 [229,230]. In humans with severe obesity, ET that improved glucose tolerance markedly reduced inflammation and macrophage infiltration in adipose tissue but not in skeletal muscle [231]. This is different to what is observed after an acute bout of exercise, where an increased infiltration of macrophages and an increase in genes related to inflammation is observed in human adipose tissue [232]. This suggests that adipose tissue and not skeletal muscle is a major contributor to the attenuation of whole-body chronic inflammation. While exercise can restore whole-body chronic inflammation, an acute bout of exercise also results in the release of IL-6 from skeletal muscle during exercise [233]. This transient elevation of IL-6 seems beneficial, as it promotes skeletal muscle glucose uptake and, as described below in the context of cancer, might help the immune system inhibit tumor growth [234].

#### Exercise Lowers Chronic Inflammation in the Context of Cancer

During high intensity and long-duration exercise, there is an acute up-regulation of IL-6 release, which might increase the infiltration of natural killer cells in cancer tissue and thereby decrease tumor growth, as has been shown in rodents [234]. In contrast to this beneficial acute spike in cytokines, a meta-analysis of current literature shows that long-term ET results in decreased systemic inflammation in cancer survivors evidenced by reduced C-reactive protein and TNF-α, with the strongest effects in breast and prostate cancer [235]. However, these data are inconclusive, as other studies report no improvement with ET on inflammation in patients with prostate cancer despite many other improvements [168,204]. A recent published study in patients treated with chemotherapy for breast cancer [236] showed that chemotherapy leads to an inflammatory environment, while a mix of resistance ET and high-intensity aerobic interval training can reduce the chemotherapy-induced inflammation and subsequent fatigue [236]. Similarly, in a phase II trial, aerobic ET (220 min/week for 12 weeks) after treatment for breast or colorectal cancer reduced the circulating levels of IL-6 with no additive effect of the anti-diabetic drug metformin in patients [237]. In rodent cancer models, aerobic ET has also been shown to decrease plasma IL-6 concentrations [238] and the cytokine content of adipose tissue [63]. Thus, ET is likely to treat chronic inflammation in patients as well as cancer survivors but whether this would impinge on metabolic function is unknown.

### 4.5. Exercise Increases Skeletal Muscle Mass and Strength

ET, especially resistance ET, leads to muscle hypertrophy and improved muscle strength [239]. This could help counter cancer cachexia and benefit metabolic regulation as skeletal muscle is a vital site for postprandial glucose uptake. Accordingly, resistance ET improves glycemic control in obese and T2D patients [240,241]. The mechanisms by which ET induces muscle hypertrophy is complex and involves multiple pathways including stimulating skeletal muscle protein synthesis, decreasing markers of protein degradation and the proteasome system (atrogin-1 and MuRF-1), reducing autophagy, and increasing mTORC1-S6K signaling, processes that have been extensively reviewed in [242,243,244].

#### Exercise Enhances Muscle Mass and Strength in the Context of Cancer

The effects of ET on muscle mass and strength in cancer cachexia is an emerging field because cancer causes negative net protein balance, while exercise could prevent this. A priority has been to establish the safety of exercise for the patients. A recent clinical trial in patients with cachectic lung and pancreatic cancer showed that a multimodal intervention, including physical activity, did not cause adverse events or poorer survival [245]. While the patients receiving the usual treatment lost body weight during the intervention period, patients receiving the multimodal intervention did not lose weight [245]. Therefore, regular exercise has potential to prevent cancer cachexia. Meanwhile, aerobic and resistance ET during and after chemo- and radiotherapy have been shown to increase lean body mass [246,247], maximal skeletal muscle strength [173,246,248,249], and myofiber cross-sectional area [173,225], while also preventing a decline in or even increasing VO_2_ peak [247,248,249,250]. Accordingly, a systematic review by Stene *et al*. showed that in patients with cancer, both aerobic and resistance ET, and a combination of these, improved upper and lower muscle strength compared to usual care [251]. Another more recent meta-analysis confirmed that muscle strength can be increased by resistance training in older patients with cancer, although muscle mass was not increased [252].

Mechanistic insight has mainly been obtained from preclinical models of cancer in which ET also increases muscle mass and strength [63,97,221,238,253,254,255,256]. ET was found to improve skeletal muscle protein synthesis [257], decrease markers of protein degradation and the proteasome system (atrogin-1 and MuRF-1) [222,253], reduce markers of autophagy [97,221,222,257], and increase mTORC1-S6K signaling [224,253,255,257]. Based on those studies, the molecular machinery for exercise to increase muscle mass and strength seem to be intact in cancer supporting a role for exercise as a potent treatment for cancer-associated cachexia. Yet, presently there is a lack of molecular knowledge in humans related to the mechanisms that underlie cancer cachexia as well as ET-induced muscle mass regulation. It is also unknown whether an acute bout of exercise might increase anabolic sensitivity in people diagnosed with cancer, as it does for healthy subjects [258,259] and elderly men [259], which could have implications for the timing of meals for patients with cachexia. Furthermore, whether acute exercise or ET would improve metabolic regulation in cachexia is unresolved.

## 5. Unanswered Questions

The following knowledge gaps hinder optimal treatment of metabolic dysfunction in cancer and hamper our ability to harness the beneficial effects of exercise:

1. The optimal exercise regimen for benefitting metabolic regulation in cancer remains to be established.
2. The appropriate implementation of exercise into the oncological treatment of cancer must be determined.
3. It is important to establish whether an acute exercise bout fully stimulates insulin-independent glucose uptake into the exercising muscles in cancer patients. This will be vital information in the daily life for cancer patients, as improved glycemic control is associated with the effectiveness of cancer treatment and improved survival.
4. It would have real-life patient benefits to determine whether the insulin-sensitizing effect of one bout of exercise exists in patients with cancer and can be exploited in relation to the timing between exercise and meals to maximize glucose disposal, reduce hyperinsulinemia, and elevate muscle protein synthesis.
5. For cancer patients with cancer cachexia, it is important to determine whether exercise can treat the loss of muscle mass and improve strength and which exercise regimen is most effective.
6. Deeper knowledge of the molecular mechanisms by which exercise benefits metabolic regulation is needed to identify novel therapeutically drug targets. This is especially important in patients with cancer cachexia who are unlikely to easily exercise [260].

## 6. Conclusions

The evidence is clear that dysregulated metabolism is a common feature of cancer. It manifests as peripheral insulin resistance, hyperlipidemia, mitochondrial dysfunction, inflammation, and cachexia. Contemporary observational studies have shown that cancer survival depends on better metabolism management strategies, as metabolic dysfunctions are associated with reduced survival and increased cancer recurrence risks for most cancers. Exercise produces acute as well as longer-term metabolic benefits by improving insulin sensitivity, restoring hyperlipidemia, reducing inflammation, enhancing mitochondrial function, and increasing muscle mass and strength. Thus, exercise could be an important strategy to improve metabolic function in cancer but randomized controlled trials in patients with cancer are warranted. The American College of Sports Medicine updated its exercise guidelines for cancer treatment of a variety of cancer health-related outcomes including fatigue, anxiety, depression, sleep, function, and quality of life [261]. Improved metabolic regulation could likely be added to that list in the future, yet, current evidence is limited and many exciting discoveries lie ahead.

## References

[1] Rohdenburg G.L., Bernhard A., Krehbiel O. (1919). Sugar tolerance in cancer. J. Am. Med. Assoc..

[2] Jasani B., Donaldson L.J., Ratcliffe J.G., Sokhi G.S. (1978). Mechanism of impaired glucose tolerance in patients with neoplasia. Br. J. Cancer.

[3] Bishop J.S., Marks P.A. (1959). Studies on carbohydrate metabolism in patients with neoplastic disease. II. Response to insulin administration. J. Clin. Investig..

[4] Luque R.M., López-Sánchez L.M., Villa-Osaba A., Luque I.M., Santos-Romero A.L., Yubero-Serrano E.M., Cara-García M., Álvarez-Benito M., López-Miranda J., Gahete M.D. (2017). Breast cancer is associated to impaired glucose/insulin homeostasis in premenopausal obese/overweight patients. Oncotarget.

[5] Permert J., Adrian T.E., Jacobsson P., Jorfelt L., Fruin A.B., Larsson J. (1993). Is profound peripheral insulin resistance in patients with pancreatic cancer caused by a tumor-associated factor?. Am. J. Surg..

[6] Winter A., MacAdams J., Chevalier S. (2012). Normal protein anabolic response to hyperaminoacidemia in insulin-resistant patients with lung cancer cachexia. Clin. Nutr..

[7] Yoshikawa T., Noguchi Y., Matsumoto A. (1994). Effects of tumor removal and body weight loss on insulin resistance in patients with cancer. Surgery.

[8] Yoshikawa T., Noguchi Y., Doi C., Makino T., Nomura K. (2001). Insulin resistance in patients with cancer: Relationships with tumor site, tumor stage, body-weight loss, acute-phase response, and energy expenditure. Nutrition.

[9] Pisters P.W., Cersosimo E., Rogatko A., Brennan M.F. (1992). Insulin action on glucose and branched-chain amino acid metabolism in cancer cachexia: Differential effects of insulin. Surgery.

[10] Copeland G.P., Leinster S.J., Davis J.C., Hipkin L.J. (1987). Insulin resistance in patients with colorectal cancer. Br. J. Surg..

[11] Lipscombe L.L., Chan W.W., Yun L., Austin P.C., Anderson G.M., Rochon P.A. (2013). Incidence of diabetes among postmenopausal breast cancer survivors. Diabetologia.

[12] Singh S., Earle C.C., Bae S.J., Fischer H.D., Yun L., Austin P.C., Rochon P.A., Anderson G.M., Lipscombe L. (2016). Incidence of Diabetes in Colorectal Cancer Survivors. J. Natl. Cancer Inst..

[13] Schoen R.E., Tangen C.M., Kuller L.H., Burke G.L., Cushman M., Tracy R.P., Dobs A., Savage P.J. (1999). Increased blood glucose and insulin, body size, and incident colorectal cancer. J. Natl. Cancer Inst..

[14] Stattin P., Björ O., Ferrari P., Lukanova A., Lenner P., Lindahl B., Hallmans G., Kaaks R. (2007). Prospective study of hyperglycemia and cancer risk. Diabetes Care.

[15] Giovannucci E., Michaud D. (2007). The Role of Obesity and Related Metabolic Disturbances in Cancers of the Colon, Prostate, and Pancreas. Gastroenterology.

[16] Rapp K., Schroeder J., Klenk J., Ulmer H., Concin H., Diem G., Oberaigner W., Weiland S.K. (2006). Fasting blood glucose and cancer risk in a cohort of more than 140,000 adults in Austria. Diabetologia.

[17] Renehan A.G., Tyson M., Egger M., Heller R.F., Zwahlen M. (2008). Body-mass index and incidence of cancer: A systematic review and meta-analysis of prospective observational studies. Lancet.

[18] Saydah S.H., Loria C.M., Eberhardt M.S., Brancati F.L. (2003). Abnormal glucose tolerance and the risk of cancer death in the United States. Am. J. Epidemiol..

[19] Sun H.J., Ohrr H., Sull J.W., Yun J.E., Ji M., Samet J.M. (2005). Fasting serum glucose level and cancer risk in Korean men and women. J. Am. Med. Assoc..

[20] Bhaskaran K., Douglas I., Forbes H., Dos-Santos-Silva I., Leon D.A., Smeeth L. (2014). Body-mass index and risk of 22 specific cancers: A population-based cohort study of 5·24 million UK adults. Lancet.

[21] Calle E.E., Rodriguez C., Walker-Thurmond K., Thun M.J. (2003). Overweight, Obesity, and Mortality from Cancer in a Prospectively Studied Cohort of U.S. Adults. N. Engl. J. Med..

[22] Campbell P.T., Newton C.C., Patel A.V., Jacobs E.J., Gapstur S.M. (2012). Diabetes and cause-specific mortality in a prospective cohort of one million U.S. adults. Diabetes Care.

[23] Harding J.L., Andes L.J., Gregg E.W., Cheng Y.J., Weir H.K., Bullard K.M., Burrows N.R., Imperatore G. (2020). Trends in cancer mortality among people with vs without diabetes in the USA, 1988-2015. Diabetologia.

[24] Calip G.S., Malone K.E., Gralow J.R., Stergachis A., Hubbard R.A., Boudreau D.M. (2014). Metabolic syndrome and outcomes following early-stage breast cancer. Breast Cancer Res. Treat..

[25] Currie C.J., Poole C.D., Jenkins-Jones S., Gale E.A.M., Johnson J.A., Morgan C.L. (2012). Mortality after incident cancer in people with and without type 2 diabetes: Impact of metformin on survival. Diabetes Care.

[26] Bowker S.L., Majumdar S.R., Veugelers P., Johnson J.A. (2006). Increased cancer-related mortality for patients with type 2 diabetes who use sulfonylureas or insulin. Diabetes Care.

[27] Pearson-Stuttard J., Bennett J., Cheng Y.J., Vamos E.P., Cross A.J., Ezzati M., Gregg E.W. (2021). Trends in predominant causes of death in individuals with and without diabetes in England from 2001 to 2018: An epidemiological analysis of linked primary care records. Lancet Diabetes Endocrinol..

[28] Mills K.T., Bellows C.F., Hoffman A.E., Kelly T.N., Gagliardi G. (2013). Diabetes mellitus and colorectal cancer prognosis: A meta-analysis. Dis. Colon Rectum.

[29] Schmitz K.H., Campbell A.M., Stuiver M.M., Pinto B.M., Schwartz A.L., Morris G.S., Ligibel J.A., Cheville A., Galvão D.A., Alfano C.M. (2019). Exercise is medicine in oncology: Engaging clinicians to help patients move through cancer. CA Cancer J. Clin..

[30] Friedenreich C.M., Neilson H.K., Farris M.S., Courneya K.S. (2016). Physical activity and cancer outcomes: A precision medicine approach. Clin. Cancer Res..

[31] DeFronzo R.A., Gunnarsson R., Björkman O., Olsson M., Wahren J. (1985). Effects of insulin on peripheral and splanchnic glucose metabolism in noninsulin-dependent (type II) diabetes mellitus. J. Clin. Investig..

[32] Ferrannini E., Bjorkman O., Reichard G.A., Pilo A., Olsson M., Wahren J., DeFronzo R.A. (1985). The disposal of an oral glucose load in healthy subjects. A quantitative study. Diabetes.

[33] Booth F.W., Roberts C.K., Laye M.J. (2012). Lack of exercise is a major cause of chronic diseases. Compr. Physiol..

[34] Neufer P.D., Bamman M.M., Muoio D.M., Bouchard C., Cooper D.M., Goodpaster B.H., Booth F.W., Kohrt W.M., Gerszten R.E., Mattson M.P. (2015). Understanding the Cellular and Molecular Mechanisms of Physical Activity-Induced Health Benefits. Cell Metab..

[35] Sylow L., Tokarz V., Richter E.A., Klip A. (2021). The many actions of insulin in skeletal muscle, the paramount tissue determining glycemia. Cell Metab..

[36] Röder P.V., Wu B., Liu Y., Han W. (2016). Pancreatic regulation of glucose homeostasis. Exp. Mol. Med..

[37] Warburg O. (1925). The metabolism of carcinoma cells 1. J. Cancer Res..

[38] Ye H., Adane B., Khan N., Alexeev E., Nusbacher N., Minhajuddin M., Stevens B.M., Winters A.C., Lin X., Ashton J.M. (2018). Subversion of Systemic Glucose Metabolism as a Mechanism to Support the Growth of Leukemia Cells. Cancer Cell.

[39] Vigneri R., Sciacca L., Vigneri P. (2020). Rethinking the Relationship between Insulin and Cancer. Trends Endocrinol. Metab..

[40] Han X., Raun S.H., Carlsson M., Sjøberg K.A., Henriquez-Olguín C., Ali M., Lundsgaard A., Fritzen A.M., Møller L.L.V., Li Z. (2020). Cancer causes metabolic perturbations associated with reduced insulin-stimulated glucose uptake in peripheral tissues and impaired muscle microvascular perfusion. Metabolism.

[41] Asp M.L., Tian M., Wendel A.A., Belury M.A. (2010). Evidence for the contribution of insulin resistance to the development of cachexia in tumor-bearing mice. Int. J. cancer.

[42] Lang C.H., Skrepnik N., Dobrescu C., Burns A.H. (2017). Impairment of insulin action on peripheral glucose uptake and hepatic glucose production in tumor-bearing rats. Am. J. Physiol. Integr. Comp. Physiol..

[43] Puppa M.J., White J.P., Sato S., Cairns M., Baynes J.W., Carson J.A. (2011). Gut barrier dysfunction in the ApcMin/+ mouse model of colon cancer cachexia. Biochim. Biophys. Acta Mol. Basis Dis..

[44] Keske M.A., Clerk L.H., Price W.J., Jahn L.A., Barrett E.J. (2009). Obesity Blunts Microvascular Recruitment in Human Forearm Muscle After a Mixed Meal. Diabetes Care.

[45] Sylow L., Jensen T.E., Kleinert M., Højlund K., Kiens B., Wojtaszewski J., Prats C., Schjerling P., Richter E.A. (2013). Rac1 signaling is required for insulin-stimulated glucose uptake and is dysregulated in insulin-resistant murine and human skeletal muscle. Diabetes.

[46] Jaiswal N., Gavin M.G., Quinn W.J., Luongo T.S., Gelfer R.G., Baur J.A., Titchenell P.M. (2019). The role of skeletal muscle Akt in the regulation of muscle mass and glucose homeostasis. Mol. Metab..

[47] Sylow L., Kleinert M., Pehmøller C., Prats C., Chiu T.T., Klip A., Richter E.A., Jensen T.E. (2014). Akt and Rac1 signaling are jointly required for insulin-stimulated glucose uptake in skeletal muscle and downregulated in insulin resistance. Cell. Signal..

[48] Chadt A., Immisch A., De Wendt C., Springer C., Zhou Z., Stermann T., Holman G.D., Loffing-Cueni D., Loffing J., Joost H.G. (2015). Deletion of both rab-GTPase-activating proteins TBC1D1 and TBC1D4 in mice eliminates insulin- and AICAR-stimulated glucose transport. Diabetes.

[49] Timmers S., De Vogel-Van Den Bosch J., Towler M.C., Schaart G., Moonen-Kornips E., Mensink R.P., Hesselink M.K., Hardie G.D., Schrauwen P. (2010). Prevention of high-fat diet-induced muscular lipid accumulation in rats by α lipoic acid is not mediated by ampk activation. J. Lipid Res..

[50] Kim Y.B., Nikoulina S.E., Ciaraldi T.P., Henry R.R., Kahn B.B. (1999). Normal insulin-dependent activation of Akt/protein kinase B, with diminished activation of phosphoinositide 3-kinase, in muscle in type 2 diabetes. J. Clin. Investig..

[51] Shao J., Yamashita H., Qiao L., Friedman J. (2000). Decreased Akt kinase activity and insulin resistance C57BL/KsJ-Lepr(db/db) mice. J. Endocrinol..

[52] Karlsson H.K.R., Zierath J.R., Kane S., Krook A., Lienhard G.E., Wallberg-Henriksson H. (2005). Insulin-stimulated phosphorylation of the Akt substrate AS160 is impaired in skeletal muscle of type 2 diabetic subjects. Diabetes.

[53] Leij-Halfwerk S., Dagnelie P.C., Van Den Berg J.W.O., Wattimena J.D.L., Hordijk-Luijk C.H., Wilson J.H.P. (2000). Weight loss and elevated gluconeogenesis from alanine in lung cancer patients. Am. J. Clin. Nutr..

[54] Leij-Halfwerk S., Van Den Berg J.W.O., Sijens P.E., Wilson J.H.P., Oudkerk M., Dagnelie P.C. (2000). Altered hepatic gluconeogenesis during L-alanine infusion in weight- losing lung cancer patients as observed by phosphorus magnetic resonance spectroscopy and turnover measurements. Cancer Res..

[55] Goncalves M.D., Hwang S.-K., Pauli C., Murphy C.J., Cheng Z., Hopkins B.D., Wu D., Loughran R.M., Emerling B.M., Zhang G. (2018). Fenofibrate prevents skeletal muscle loss in mice with lung cancer. Proc. Natl. Acad. Sci. USA.

[56] Pötgens S.A., Thibaut M.M., Joudiou N., Sboarina M., Neyrinck A.M., Cani P.D., Claus S.P., Delzenne N.M., Bindels L.B. (2021). Multi-compartment metabolomics and metagenomics reveal major hepatic and intestinal disturbances in cancer cachectic mice. J. Cachexia Sarcopenia Muscle.

[57] Raza U., Asif M.R., Bin Rehman A., Sheikh A. (2018). Hyperlipidemia and hyper glycaemia in Breast Cancer Patients is related to disease stage. Pakistan J. Med. Sci..

[58] Xie C., Wen P., Su J., Li Q., Ren Y., Liu Y., Shen R., Ren J. (2019). Elevated serum triglyceride and low-density lipoprotein cholesterol promotes the formation of colorectal polyps. BMC Gastroenterol..

[59] Shah F.D., Shukla S.N., Shah P.M., Patel H.R.H. (2008). Prabhudas Shankerbhai Patel Significance of Alterations in Plasma Lipid Profile Levels in Breast Cancer. Integr. Cancer Ther..

[60] Fiorenza A.M., Branchi A., Sommariva D. (2000). Serum lipoprotein profile in patients with cancer. A comparison with non-cancer subjects. Int. J. Clin. Lab. Res..

[61] Das S.K., Eder S., Schauer S., Diwoky C., Temmel H., Guertl B., Gorkiewicz G., Tamilarasan K.P., Kumari P., Trauner M. (2011). Adipose triglyceride lipase contributes to cancer-associated cachexia. Science.

[62] López-Soriano J., Llovera M., Carbó N., García-Martínez C., López-Soriano F.J., Argiles J.M. (1997). Lipid metabolism in tumour-bearing mice: Studies with knockout mice for tumour necrosis factor receptor 1 protein. Mol. Cell. Endocrinol..

[63] Donatto F.F., Neves R.X., Rosa F.O., Camargo R.G., Ribeiro H., Matos-Neto E.M., Seelaender M. (2013). Resistance exercise modulates lipid plasma profile and cytokine content in the adipose tissue of tumour-bearing rats. Cytokine.

[64] Huang J., Li L., Lian J., Schauer S., Vesely P.W., Kratky D., Hoefler G., Lehner R. (2016). Tumor-Induced Hyperlipidemia Contributes to Tumor Growth. Cell Rep..

[65] O’Connell T.M., Ardeshirpour F., Asher S.A., Winnike J.H., Yin X., George J., Guttridge D.C., He W., Wysong A., Willis M.S. (2008). Metabolomic analysis of cancer cachexia reveals distinct lipid and glucose alterations. Metabolomics.

[66] Silvério R., Lira F.S., Oyama L.M., Oller Do Nascimento C.M., Otoch J.P., Alcântara P.S.M., Batista M.L., Seelaender M. (2017). Lipases and lipid droplet-associated protein expression in subcutaneous white adipose tissue of cachectic patients with cancer. Lipids Health Dis..

[67] Manley G. (2013). Public Access NIH Public Access. Hear. Metab..

[68] Hoeg L.D., Sjoberg K.A., Jeppesen J., Jensen T.E., Frosig C., Birk J.B., Bisiani B., Hiscock N., Pilegaard H., Wojtaszewski J.F.P. (2011). Lipid-induced insulin resistance affects women less than men and is not accompanied by inflammation or impaired proximal insulin signaling. Diabetes.

[69] Reaven G.M., Hollenbeck C., Jeng C.Y., Wu M.S., Chen Y.D.I. (1988). Measurement of plasma glucose, free fatty acid, lactate, and insulin for 24 h in patients with NIDDM. Diabetes.

[70] Bódis K., Roden M. (2018). Energy metabolism of white adipose tissue and insulin resistance in humans. Eur. J. Clin. Investig..

[71] Morigny P., Zuber J., Haid M., Kaltenecker D., Riols F., Lima J.D.C., Simoes E., Otoch J.P., Schmidt S.F., Herzig S. (2020). High levels of modified ceramides are a defining feature of murine and human cancer cachexia. J. Cachexia Sarcopenia Muscle.

[72] Stephens N.A., Skipworth R.J.E., MacDonald A.J., Greig C.A., Ross J.A., Fearon K.C.H. (2011). Intramyocellular lipid droplets increase with progression of cachexia in cancer patients. J. Cachexia Sarcopenia Muscle.

[73] Fukawa T., Yan-Jiang B.C., Min-Wen J.C., Jun-Hao E.T., Huang D., Qian C.N., Ong P., Li Z., Chen S., Mak S.Y. (2016). Excessive fatty acid oxidation induces muscle atrophy in cancer cachexia. Nat. Med..

[74] Diaz M.B., Krones-Herzig A., Metzger D., Ziegler A., Vegiopoulos A., Klingenspor M., Müller-Decker K., Herzig S. (2008). Nuclear receptor cofactor receptor interacting protein 140 controls hepatic triglyceride metabolism during wasting in mice. Hepatology.

[75] Petersen M.C., Shulman G.I. (2018). Mechan isms of insulin action and insulin resistance. Physiol. Rev..

[76] Fazakerley D.J., Krycer J.R., Kearney A.L., Hocking S.L., James D.E. (2019). Muscle and adipose tissue insulin resistance: Malady without mechanism?. J. Lipid Res..

[77] Romanello V., Sandri M. (2016). Mitochondrial quality control and muscle mass maintenance. Front. Physiol..

[78] Ebhardt H.A., Degen S., Tadini V., Schilb A., Johns N., Greig C.A., Fearon K.C.H., Aebersold R., Jacobi C. (2017). Comprehensive proteome analysis of human skeletal muscle in cachexia and sarcopenia: A pilot study. J. Cachexia Sarcopenia Muscle.

[79] Wilson H.E., Stanton D.A., Montgomery C., Infante A.M., Taylor M., Hazard-Jenkins H., Pugacheva E.N., Pistilli E.E. (2020). Skeletal muscle reprogramming by breast cancer regardless of treatment history or tumor molecular subtype. npj Breast Cancer.

[80] Barreto R., Mandili G., Witzmann F.A., Novelli F., Zimmers T.A., Bonetto A. (2016). Cancer and chemotherapy contribute to muscle loss by activating common signaling pathways. Front. Physiol..

[81] Shum A.M.Y., Poljak A., Bentley N.L., Turner N., Tan T.C., Polly P. (2018). Proteomic profiling of skeletal and cardiac muscle in cancer cachexia: Alterations in sarcomeric and mitochondrial protein expression. Oncotarget.

[82] Kunzke T., Buck A., Prade V.M., Feuchtinger A., Prokopchuk O., Martignoni M.E., Heisz S., Hauner H., Janssen K., Walch A. (2020). Derangements of amino acids in cachectic skeletal muscle are caused by mitochondrial dysfunction. J. Cachexia Sarcopenia Muscle.

[83] Montero-Bullon J.-F., Melo T., Ferreira R., Padrão A.I., Oliveira P.A., Domingues M.R.M., Domingues P. (2019). Exercise training counteracts urothelial carcinoma-induced alterations in skeletal muscle mitochondria phospholipidome in an animal model. Sci. Rep..

[84] Brown J.L., Rosa-Caldwell M.E., Lee D.E., Blackwell T.A., Brown L.A., Perry R.A., Haynie W.S., Hardee J.P., Carson J.A., Wiggs M.P. (2017). Mitochondrial degeneration precedes the development of muscle atrophy in progression of cancer cachexia in tumour-bearing mice. J. Cachexia Sarcopenia Muscle.

[85] Fontes-Oliveira C.C., Busquets S., Toledo M., Penna F., Aylwin M.P., Sirisi S., Silva A.P., Orpí M., García A., Sette A. (2013). Mitochondrial and sarcoplasmic reticulum abnormalities in cancer cachexia: Altered energetic efficiency?. Biochim. Biophys. Acta - Gen. Subj..

[86] Halle J.L., Pena G.S., Paez H.G., Castro A.J., Rossiter H.B., Visavadiya N.P., Whitehurst M.A., Khamoui A.V. (2019). Tissue-specific dysregulation of mitochondrial respiratory capacity and coupling control in colon-26 tumor-induced cachexia. Am. J. Physiol. Integr. Comp. Physiol..

[87] Shum A.M.Y., Mahendradatta T., Taylor R.J., Painter A.B., Moore M.M., Tsoli M., Tan T.C., Clarke S.J., Robertson G.R., Polly P. (2012). Disruption of MEF2C signaling and loss of sarcomeric and mitochondrial integrity in cancer-induced skeletal muscle wasting. Aging.

[88] Hardee J.P., Mangum J.E., Gao S., Sato S., Hetzler K.L., Puppa M.J., Fix D.K., Carson J.A. (2016). Eccentric contraction-induced myofiber growth in tumor-bearing mice. J. Appl. Physiol..

[89] White J.P., Puppa M.J., Sato S., Gao S., Price R.L., Baynes J.W., Kostek M.C., Matesic L.E., Carson J.A. (2012). IL-6 regulation on skeletal muscle mitochondrial remodeling during cancer cachexia in the ApcMin/+ mouse. Skelet. Muscle.

[90] Figueroa-Clarevega A., Bilder D. (2015). Malignant drosophila tumors interrupt insulin signaling to induce cachexia-like wasting. Dev. Cell.

[91] de Castro G.S., Simoes E., Lima J.D.C.C., Ortiz-Silva M., Festuccia W.T., Tokeshi F., Alcântara P.S., Otoch J.P., Coletti D., Seelaender M. (2019). Human Cachexia Induces Changes in Mitochondria, Autophagy and Apoptosis in the Skeletal Muscle. Cancers.

[92] Guigni B.A., Callahan D.M., Tourville T.W., Miller M.S., Fiske B., Voigt T., Korwin-Mihavics B., Anathy V., Dittus K., Toth M.J. (2018). Skeletal muscle atrophy and dysfunction in breast cancer patients: Role for chemotherapy-derived oxidant stress. Am. J. Physiol. Physiol..

[93] Green K., Brand M.D., Murphy M.P. (2004). Prevention of Mitochondrial Oxidative Damage As A Therapeutic Strategy in Diabetes. Diabetes.

[94] Koves T.R., Ussher J.R., Noland R.C., Slentz D., Mosedale M., Ilkayeva O., Bain J., Stevens R., Dyck J.R.B., Newgard C.B. (2008). Mitochondrial Overload and Incomplete Fatty Acid Oxidation Contribute to Skeletal Muscle Insulin Resistance. Cell Metab..

[95] Alves C.R.R., das Neves W., de Almeida N.R., Eichelberger E.J., Jannig P.R., Voltarelli V.A., Tobias G.C., Bechara L.R.G., de Paula Faria D., Alves M.J.N. (2020). Exercise training reverses cancer-induced oxidative stress and decrease in muscle COPS2/TRIP15/ALIEN. Mol. Metab..

[96] Padilha C.S., Borges F.H., Costa Mendes da Silva L.E., Frajacomo F.T.T., Jordao A.A., Duarte J.A., Cecchini R., Guarnier F.A., Deminice R. (2017). Resistance exercise attenuates skeletal muscle oxidative stress, systemic pro-inflammatory state, and cachexia in Walker-256 tumor-bearing rats. Appl. Physiol. Nutr. Metab..

[97] Ballarò R., Penna F., Pin F., Gómez-Cabrera M., Viña J., Costelli P. (2019). Moderate Exercise Improves Experimental Cancer Cachexia by Modulating the Redox Homeostasis. Cancers.

[98] Dumas J.-F., Goupille C., Julienne C.M., Pinault M., Chevalier S., Bougnoux P., Servais S., Couet C. (2011). Efficiency of oxidative phosphorylation in liver mitochondria is decreased in a rat model of peritoneal carcinosis. J. Hepatol..

[99] Khamoui A.V., Tokmina-Roszyk D., Rossiter H.B., Fields G.B., Visavadiya N.P. (2020). Hepatic proteome analysis reveals altered mitochondrial metabolism and suppressed acyl-CoA synthetase-1 in colon-26 tumor-induced cachexia. Physiol. Genomics.

[100] Visavadiya N.P., Pena G.S., Khamoui A.V. (2020). Mitochondrial dynamics and quality control are altered in a hepatic cell culture model of cancer cachexia. Mol. Cell. Biochem..

[101] Petruzzelli M., Schweiger M., Schreiber R., Campos-Olivas R., Tsoli M., Allen J., Swarbrick M., Rose-John S., Rincon M., Robertson G. (2014). A Switch from White to Brown Fat Increases Energy Expenditure in Cancer-Associated Cachexia. Cell Metab..

[102] Becker A.S., Zellweger C., Bacanovic S., Franckenberg S., Nagel H.W., Frick L., Schawkat K., Eberhard M., Blüthgen C., Volbracht J. (2020). Brown fat does not cause cachexia in cancer patients: A large retrospective longitudinal FDG-PET/CT cohort study. PLoS One.

[103] Miller J., Dreczkowski G., Ramage M.I., Wigmore S.J., Gallagher I.J., Skipworth R.J.E. (2020). Adipose depot gene expression and intelectin-1 in the metabolic response to cancer and cachexia. J. Cachexia Sarcopenia Muscle.

[104] Scott H.R., McMillan D.C., Crilly A., McArdle C.S., Milroy R. (1996). The relationship between weight loss and interleukin 6 in non-small-cell lung cancer. Br. J. Cancer.

[105] Onesti J.K., Guttridge D.C. (2014). Inflammation Based Regulation of Cancer Cachexia. Biomed Res. Int..

[106] Saltiel A.R., Olefsky J.M. (2017). Inflammatory mechanisms linking obesity and metabolic disease. J. Clin. Investig..

[107] Haddad F., Zaldivar F., Cooper D.M., Adams G.R. (2005). IL-6-induced skeletal muscle atrophy. J. Appl. Physiol..

[108] Goodman M.N. (1991). Tumor necrosis factor induces skeletal muscle protein breakdown in rats. Am. J. Physiol.-Endocrinol. Metab..

[109] Strassmann G., Fong M., Kenney J.S., Jacob C.O. (1992). Evidence for the involvement of interleukin 6 in experimental cancer cachexia. J. Clin. Investig..

[110] White J.P., Baynes J.W., Welle S.L., Kostek M.C., Matesic L.E., Sato S., Carson J.A. (2011). The Regulation of Skeletal Muscle Protein Turnover during the Progression of Cancer Cachexia in the ApcMin/+ Mouse. PLoS One.

[111] Baltgalvis K.A., Berger F.G., Pena M.M.O., Davis J.M., Muga S.J., Carson J.A. (2008). Interleukin-6 and cachexia in ApcMin/+ mice. Am. J. Physiol..

[112] Prado B.L., Qian Y. (2019). Anti-cytokines in the treatment of cancer cachexia. Ann. Palliat. Med..

[113] Fearon K., Strasser F., Anker S.D., Bosaeus I., Bruera E., Fainsinger R.L., Jatoi A., Loprinzi C., MacDonald N., Mantovani G. (2011). Definition and classification of cancer cachexia: An international consensus. Lancet Oncol..

[114] Christensen J.F., Jones L.W., Andersen J.L., Daugaard G., Rorth M., Hojman P. (2014). Muscle dysfunction in cancer patients. Ann. Oncol..

[115] O’Neill B.T., Lee K.Y., Klaus K., Softic S., Krumpoch M.T., Fentz J., Stanford K.I., Robinson M.M., Cai W., Kleinridders A. (2016). Insulin and IGF-1 receptors regulate FoxO-mediated signaling in muscle proteostasis. J. Clin. Investig..

[116] Ebner N., Anker S.D., von Haehling S. (2020). Recent developments in the field of cachexia, sarcopenia, and muscle wasting: Highlights from the 12th Cachexia Conference. J. Cachexia Sarcopenia Muscle.

[117] Dolly A., Dumas J.F., Servais S. (2020). Cancer cachexia and skeletal muscle atrophy in clinical studies: What do we really know?. J. Cachexia Sarcopenia Muscle.

[118] Argilés J.M., Stemmler B., López-Soriano F.J., Busquets S. (2019). Inter-tissue communication in cancer cachexia. Nat. Rev. Endocrinol..

[119] Biswas A.K., Acharyya S. (2020). Understanding cachexia in the context of metastatic progression. Nat. Rev. Cancer.

[120] Cohen S., Nathan J.A., Goldberg A.L. (2015). Muscle wasting in disease: Molecular mechanisms and promising therapies. Nat. Rev. Drug Discov..

[121] Sandri M. (2016). Protein breakdown in cancer cachexia. Semin. Cell Dev. Biol..

[122] Emery P.W., Edwards R.H., Rennie M.J., Souhami R.L., Halliday D. (1984). Protein synthesis in muscle measured in vivo in cachectic patients with cancer. BMJ.

[123] Brown J.L., Lee D.E., Rosa-Caldwell M.E., Brown L.A., Perry R.A., Haynie W.S., Huseman K., Sataranatarajan K., Van Remmen H., Washington T.A. (2018). Protein imbalance in the development of skeletal muscle wasting in tumour-bearing mice. J. Cachexia Sarcopenia Muscle.

[124] Lima M., Sato S., Enos R.T., Baynes J.W., Carson J.A. (2013). Development of an UPLC mass spectrometry method for measurement of myofibrillar protein synthesis: Application to analysis of murine muscles during cancer cachexia. J. Appl. Physiol..

[125] Smith K., Tisdale M. (1993). Increased protein degradation and decreased protein synthesis in skeletal muscle during cancer cachexia. Br. J. Cancer.

[126] Murton A.J., Maddocks M., Stephens F.B., Marimuthu K., England R., Wilcock A. (2017). Consequences of Late-Stage Non–Small-Cell Lung Cancer Cachexia on Muscle Metabolic Processes. Clin. Lung Cancer.

[127] Op Den Kamp C.M., Langen R.C., Snepvangers F.J., De Theije C.C., Schellekens J.M., Laugs F., Dingemans A.M.C., Schols A.M. (2013). Nuclear transcription factor κB activation and protein turnover adaptations in skeletal muscle of patients with progressive stages of lung cancer cachexia. Am. J. Clin. Nutr..

[128] Puig-Vilanova E., Rodriguez D.A., Lloreta J., Ausin P., Pascual-Guardia S., Broquetas J., Roca J., Gea J., Barreiro E. (2015). Oxidative stress, redox signaling pathways, and autophagy in cachectic muscles of male patients with advanced COPD and lung cancer. Free Radic. Biol. Med..

[129] Zhang Y., Wang J., Wang X., Gao T., Tian H., Zhou D., Zhang L., Li G., Wang X. (2020). The autophagic-lysosomal and ubiquitin proteasome systems are simultaneously activated in the skeletal muscle of gastric cancer patients with cachexia. Am. J. Clin. Nutr..

[130] Srikanthan P., Karlamangla A.S. (2011). Relative muscle mass is inversely associated with insulin resistance and prediabetes. Findings from the third national health and nutrition examination survey. J. Clin. Endocrinol. Metab..

[131] Wall B.T., Gorissen S.H., Pennings B., Koopman R., Groen B.B.L., Verdijk L.B., van Loon L.J.C. (2015). Aging Is Accompanied by a Blunted Muscle Protein Synthetic Response to Protein Ingestion. PLoS One.

[132] Volpi E., Mittendorfer B., Rasmussen B.B., Wolfe R.R. (2000). The Response of Muscle Protein Anabolism to Combined Hyperaminoacidemia and Glucose-Induced Hyperinsulinemia Is Impaired in the Elderly 1. J. Clin. Endocrinol. Metab..

[133] Guillet C., Prod’homme M., Balage M., Gachon P., Giraudet C., Morin L., Grizard J., Boirie Y. (2004). Impaired anabolic response of muscle protein synthesis is associated with S6K1 dysregulation in elderly humans. FASEB J..

[134] Kwon Y., Song W., Droujinine I.A., Hu Y., Asara J.M., Perrimon N. (2015). Systemic organ wasting induced by localized expression of the secreted Insulin/IGF antagonist ImpL2. Dev. Cell.

[135] Savikj M., Zierath J.R. (2020). Train like an athlete: Applying exercise interventions to manage type 2 diabetes. Diabetologia.

[136] Sylow L., Richter E.A. (2019). Current advances in our understanding of exercise as medicine in metabolic disease. Curr. Opin. Physiol..

[137] Hargreaves M., Spriet L.L. (2020). Skeletal muscle energy metabolism during exercise. Nat. Metab..

[138] Kennedy J.W., Hirshman M.F., Gervino E.V., Ocel J.V., Forse R.A., Hoenig S.J., Aronson D., Goodyear L.J., Horton E.S. (1999). Acute exercise induces GLUT4 translocation in skeletal muscle of normal human subjects and subjects with type 2 diabetes. Diabetes.

[139] Wojtaszewski J.F.P., Higaki Y., Hirshman M.F., Michael M.D., Dufresne S.D., Kahn C.R., Goodyear L.J. (1999). Exercise modulates postreceptor insulin signaling and glucose transport in muscle-specific insulin receptor knockout mice. J. Clin. Investig..

[140] Sylow L., Kleinert M., Richter E.A., Jensen T.E. (2017). Exercise-stimulated glucose uptake-regulation and implications for glycaemic control. Nat. Rev. Endocrinol..

[141] O’Neill H.M., Maarbjerg S.J., Crane J.D., Jeppesen J., Jørgensen S.B., Schertzer J.D., Shyroka O., Kiens B., van Denderen B.J., Tarnopolsky M.A. (2011). AMP-activated protein kinase (AMPK) beta1beta2 muscle null mice reveal an essential role for AMPK in maintaining mitochondrial content and glucose uptake during exercise. Proc. Natl. Acad. Sci. USA.

[142] Jørgensen S.B., Viollet B., Andreelli F., Frøsig C., Birk J.B., Schjerling P., Vaulont S., Richter E.A., Wojtaszewski J.F.P. (2004). Knockout of the α 2 but Not α 1 5′-AMP-activated Protein Kinase Isoform Abolishes 5-Aminoimidazole-4-carboxamide-1-β-4-ribofuranosidebut Not Contraction-induced Glucose Uptake in Skeletal Muscle. J. Biol. Chem..

[143] Lee-young R.S., Griffee S.R., Lynes S.E., Bracy D.P., Ayala J.E., Mcguinness O.P., Wasserman D.H. (2009). Skeletal Muscle AMP-activated Protein Kinase Is Essential for the Metabolic Response to Exercise In Vivo. J. Biol. Chem..

[144] Sylow L., Møller L.L.V., Kleinert M., D’Hulst G., De Groote E., Schjerling P., Steinberg G.R., Jensen T.E., Richter E.A. (2017). Rac1 and AMPK Account for the Majority of Muscle Glucose Uptake Stimulated by Ex Vivo Contraction but Not In Vivo Exercise. Diabetes.

[145] Sylow L., Jensen T.E., Kleinert M., Mouatt J.R., Maarbjerg S.J., Jeppesen J., Prats C., Chiu T.T., Boguslavsky S., Klip A. (2013). Rac1 is a novel regulator of contraction-stimulated glucose uptake in skeletal muscle. Diabetes.

[146] Sylow L., Nielsen I.L., Kleinert M., Møller L.L.V., Ploug T., Schjerling P., Bilan P.J., Klip A., Jensen T.E., Richter E.A. (2016). Rac1 governs exercise-stimulated glucose uptake in skeletal muscle through regulation of GLUT4 translocation in mice. J. Physiol..

[147] Henríquez-Olguin C., Knudsen J.R., Raun S.H., Li Z., Dalbram E., Treebak J.T., Sylow L., Holmdahl R., Richter E.A., Jaimovich E. (2019). Cytosolic ROS production by NADPH oxidase 2 regulates muscle glucose uptake during exercise. Nat. Commun..

[148] Hoffman N.J., Parker B.L., Chaudhuri R., Fisher-Wellman K.H., Kleinert M., Humphrey S.J., Yang P., Holliday M., Trefely S., Fazakerley D.J. (2015). Global Phosphoproteomic Analysis of Human Skeletal Muscle Reveals a Network of Exercise-Regulated Kinases and AMPK Substrates. Cell Metab..

[149] Deshmukh A.S., Steenberg D.E., Hostrup M., Birk J.B., Larsen J.K., Santos A., Kjøbsted R., Hingst J.R., Schéele C.C., Murgia M. (2021). Deep muscle-proteomic analysis of freeze-dried human muscle biopsies reveals fiber type-specific adaptations to exercise training. Nat. Commun..

[150] Richter E.A., Garetto L.P., Goodman M.N., Ruderman N.B. (1982). Muscle glucose metabolism following exercise in the rat. Increased sensitivity to insulin. J. Clin. Investig..

[151] Richter E.A., Mikines K.J., Galbo H., Kiens B. (1989). Effect of exercise on insulin action in human skeletal muscle. J Appl Physiol.

[152] Cartee G.D., Young D.A., Sleeper M.D., Zierath J., Wallberg-Henriksson H., Holloszy J.O. (1989). Prolonged increase in insulin-stimulated glucose transport in muscle after exercise. Am. J. Physiol. Endocrinol. Metab..

[153] Wojtaszewski J.F.P., Hansen B.F., Gade J., Kiens B., Markuns J.F., Goodyear L.J., Richter E.A. (2000). Insulin signaling and insulin sensitivity after exercise in human skeletal muscle. Diabetes.

[154] Wojtaszewski J.F.P., Hansen B.F., Kiens B., Richter E.A. (1997). Insulin Signaling in Human Skeletal Muscle: Time Course and Effect of Exercise. Diabetes.

[155] Steenberg D.E., Hingst J.R., Birk J.B., Thorup A., Kristensen J.M., Sjøberg K.A., Kiens B., Richter E.A., Wojtaszewski J.F.P. (2020). A Single Bout of One-Legged Exercise to Local Exhaustion Decreases Insulin Action in Nonexercised Muscle Leading to Decreased Whole-Body Insulin Action. Diabetes.

[156] Mikines K.J., Sonne B., Farrell P.A., Tronier B., Galbo H. (1988). Effect of physical exercise on sensitivity and responsiveness to insulin in humans. Am. J. Physiol. Metab..

[157] Sjøberg K.A., Frøsig C., Kjøbsted R., Sylow L., Kleinert M., Betik A.C., Shaw C.S., Kiens B., Wojtaszewski J.F.P., Rattigan S. (2017). Exercise Increases Human Skeletal Muscle Insulin Sensitivity via Coordinated Increases in Microvascular Perfusion and Molecular Signaling. Diabetes.

[158] Kjøbsted R., Chadt A., Jørgensen N.O., Kido K., Larsen J.K., de Wendt C., Al-Hasani H., Wojtaszewski J.F.P. (2019). TBC1D4 Is Necessary for Enhancing Muscle Insulin Sensitivity in Response to AICAR and Contraction. Diabetes.

[159] Kjøbsted R., Munk-Hansen N., Birk J.B., Foretz M., Viollet B., Björnholm M., Zierath J.R., Treebak J.T., Wojtaszewski J.F.P. (2017). Enhanced Muscle Insulin Sensitivity After Contraction/Exercise Is Mediated by AMPK. Diabetes.

[160] Sylow L., Møller L.L.V., D’Hulst G., Schjerling P., Jensen T.E., Richter E.A. (2016). Rac1 in Muscle Is Dispensable for Improved Insulin Action After Exercise in Mice. Endocrinology.

[161] Mikines K.J., Richter E.A., Dela F., Galbo H. (1991). Seven days of bed rest decrease insulin action on glucose uptake in leg and whole body. J. Appl. Physiol..

[162] Richter E.A., Kiens B., Mizuno M., Strange S. (1989). Insulin action in human thighs after one-legged immobilization. J. Appl. Physiol..

[163] Mortensen B., Friedrichsen M., Andersen N.R., Alibegovic A.C., Højbjerre L., Sonne M.P., Stallknecht B., Dela F., Wojtaszewski J.F.P., Vaag A. (2014). Physical inactivity affects skeletal muscle insulin signaling in a birth weight-dependent manner. J. Diabetes Complications.

[164] Van Dijk J.W., Venema M., Van Mechelen W., Stehouwer C.D.A., Hartgens F., Van Loon L.J.C. (2013). Effect of moderate-intensity exercise versus activities of daily living on 24-hour blood glucose homeostasis in male patients with type 2 diabetes. Diabetes Care.

[165] Mikines K.J., Sonne B., Farrell P.A., Tronier B., Galbo H. (1989). Effect of training on the dose-response relationship for insulin action in men. J. Appl. Physiol..

[166] Bird S.R., Hawley J.A. (2017). Update on the effects of physical activity on insulin sensitivity in humans. BMJ Open Sport Exerc. Med..

[167] Steenberg D.E., Jørgensen N.B., Birk J.B., Sjøberg K.A., Kiens B., Richter E.A., Wojtaszewski J.F.P. (2019). Exercise training reduces the insulin-sensitizing effect of a single bout of exercise in human skeletal muscle. J. Physiol..

[168] Hvid T., Winding K., Rinnov A., Dejgaard T., Thomsen C., Iversen P., Brasso K., Mikines K.J., Van Hall G., Lindegaard B. (2013). Endurance training improves insulin sensitivity and body composition in prostate cancer patients treated with androgen deprivation therapy. Endocr. Relat. Cancer.

[169] Brown J.C., Rickels M.R., Troxel A.B., Zemel B.S., Damjanov N., Ky B., Rhim A.D., Rustgi A.K., Courneya K.S., Schmitz K.H. (2018). Dose-response effects of exercise on insulin among colon cancer survivors. Endocr. Relat. Cancer.

[170] Lee D.H., Kim J.Y., Lee M.K., Lee C., Min J.-H., Jeong D.H., Lee J.-W., Chu S.H., Meyerhardt J.A., Ligibel J. (2013). Effects of a 12-week home-based exercise program on the level of physical activity, insulin, and cytokines in colorectal cancer survivors: A pilot study. Support. Care Cancer.

[171] Dieli-Conwright C.M., Courneya K.S., Demark-Wahnefried W., Sami N., Lee K., Buchanan T.A., Spicer D.V., Tripathy D., Bernstein L., Mortimer J.E. (2018). Effects of aerobic and resistance exercise on metabolic syndrome, sarcopenic obesity, and circulating biomarkers in overweight or obese survivors of breast cancer: A randomized controlled trial. J. Clin. Oncol..

[172] Viskochil R., Blankenship J.M., Makari-Judson G., Staudenmayer J., Freedson P.S., Hankinson S.E., Braun B. (2020). Metrics of diabetes risk are only minimally improved by exercise training in postmenopausal breast cancer survivors. J. Clin. Endocrinol. Metab..

[173] Møller A.B., Lønbro S., Farup J., Voss T.S., Rittig N., Wang J., Højris I., Mikkelsen U.R., Jessen N. (2019). Molecular and cellular adaptations to exercise training in skeletal muscle from cancer patients treated with chemotherapy. J. Cancer Res. Clin. Oncol..

[174] Puppa M.J., White J.P., Velázquez K.T., Baltgalvis K.A., Sato S., Baynes J.W., Carson J.A. (2012). The effect of exercise on IL-6-induced cachexia in the ApcMin/+ mouse. J. Cachexia Sarcopenia Muscle.

[175] Sennott J., Morrissey J., Standley P.R., Broderick T.L. (2008). Treadmill exercise training fails to reverse defects in glucose, insulin and muscle GLUT4 content in the db/db mouse model of diabetes. Pathophysiology.

[176] McKie G.L., Medak K.D., Knuth C.M., Shamshoum H., Townsend L.K., Peppler W.T., Wright D.C. (2019). Housing temperature affects the acute and chronic metabolic adaptations to exercise in mice. J. Physiol..

[177] Sun Y., Cui D., Zhang Z., Zhang Q., Ji L., Ding S. (2016). Voluntary wheel exercise alters the levels of miR-494 and miR-696 in the skeletal muscle of C57BL/6 mice. Comp. Biochem. Physiol. Part B Biochem. Mol. Biol..

[178] Ritchie I.R.W., Wright D.C., Dyck D.J. (2014). Adiponectin is not required for exercise training-induced improvements in glucose and insulin tolerance in mice. Physiol. Rep..

[179] Peppler W.T., Anderson Z.G., Sutton C.D., Rector R.S., Wright D.C. (2016). Voluntary wheel running attenuates lipopolysaccharide-induced liver inflammation in mice. Am. J. Physiol..

[180] Raun S.H., Henriquez-Olguín C., Karavaeva I., Ali M., Møller L.L.V., Kot W., Castro-Mejía J.L., Nielsen D.S., Gerhart-Hines Z., Richter E.A. (2020). Housing temperature influences exercise training adaptations in mice. Nat. Commun..

[181] Stallknecht B., Dela F., Helge J.W. (2007). Are blood flow and lipolysis in subcutaneous adipose tissue influenced by contractions in adjacent muscles in humans?. Am. J. Physiol. Metab..

[182] Mulla N., Simonsen L., Bülow J. (2000). Post-exercise adipose tissue and skeletal muscle lipid metabolism in humans: The effects of exercise intensity. J. Physiol..

[183] Moro C., Crampes F., Sengenes C., De Glisezinski I., Galitzky J., Thalamas C., Lafontan M., Berlan M. (2004). Atrial natriuretic peptide contributes to the physiological control of lipid mobilization in humans. FASEB J..

[184] Stallknecht B., Lorentsen J., Enevoldsen L.H., Bülow J., Biering-Sørensen F., Galbo H., Kjær M. (2001). Role of the sympathoadrenergic system in adipose tissue metabolism during exercise in humans. J. Physiol..

[185] Wolfe R.R., Klein S., Carraro F., Weber J.M. (1990). Role of triglyceride-fatty acid cycle in controlling fat metabolism in humans during and after exercise. Am. J. Physiol. Metab..

[186] Lundsgaard A.M., Fritzen A.M., Kiens B. (2020). The importance of fatty acids as nutrients during post-exercise recovery. Nutrients.

[187] Honkala S.M., Motiani P., Kivelä R., Hemanthakumar K.A., Tolvanen E., Motiani K.K., Eskelinen J.-J., Virtanen K.A., Kemppainen J., Heiskanen M.A. (2020). Exercise training improves adipose tissue metabolism and vasculature regardless of baseline glucose tolerance and sex. BMJ Open Diabetes Res. Care.

[188] Duncan R.E., Ahmadian M., Jaworski K., Sarkadi-Nagy E., Sul H.S. (2007). Regulation of Lipolysis in Adipocytes. Annu. Rev. Nutr..

[189] Plaisance E.P., Fisher G. (2014). Exercise and Dietary-Mediated Reductions in Postprandial Lipemia. J. Nutr. Metab..

[190] Schlierf G., Dinsenbacher A., Kather H., Kohlmeier M., Haberbosch W. (1987). Mitigation of alimentary lipemia by postprandial exercise—Phenomena and mechanisms. Metabolism.

[191] Tsetsonis N.V., Hardman A.E., Mastana S.S. (1997). Acute effects of exercise on postprandial lipemia: A comparative study in trained and untrained middle-aged women. Am. J. Clin. Nutr..

[192] ZIOGAS G.G., THOMAS T.R., HARRIS W.S. (1997). Exercise training, postprandial hypertriglyceridemia, and LDL subfraction distribution. Med. Sci. Sport. Exerc..

[193] Harley Hartung G., Lawrence S.J., Reeves R.S., Foreyt J.P. (1993). Effect of alcohol and exercise on postprandial lipemia and triglyceride clearance in men. Atherosclerosis.

[194] Merrill J.R., Holly R.G., Anderson R.L., Rifai N., King M.E., DeMeersman R. (1989). Hyperlipemic response of young trained and untrained men after a high fat meal. Arteriosclerosis.

[195] Cohen J.C., Noakes T.D., Benade A.J.S. (1989). Postprandial lipemia and chylomicron clearance in athletes and in sedentary men. Am. J. Clin. Nutr..

[196] Talanian J.L., Holloway G.P., Snook L.A., Heigenhauser G.J.F., Bonen A., Spriet L.L. (2010). Exercise training increases sarcolemmal and mitochondrial fatty acid transport proteins in human skeletal muscle. Am. J. Physiol. Metab..

[197] Louche K., Badin P.-M., Montastier E., Laurens C., Bourlier V., de Glisezinski I., Thalamas C., Viguerie N., Langin D., Moro C. (2013). Endurance Exercise Training Up-Regulates Lipolytic Proteins and Reduces Triglyceride Content in Skeletal Muscle of Obese Subjects. J. Clin. Endocrinol. Metab..

[198] Alsted T.J., Nybo L., Schweiger M., Fledelius C., Jacobsen P., Zimmermann R., Zechner R., Kiens B. (2009). Adipose triglyceride lipase in human skeletal muscle is upregulated by exercise training. Am. J. Physiol. Metab..

[199] Jong-Yeon K., Hickner R.C., Dohm G.L., Houmard J.A. (2002). Long- and medium-chain fatty acid oxidation is increased in exercise-trained human skeletal muscle. Metabolism.

[200] Fritzen A.M., Lundsgaard A.-M., Kiens B. (2020). Tuning fatty acid oxidation in skeletal muscle with dietary fat and exercise. Nat. Rev. Endocrinol..

[201] Bacchi E., Negri C., Targher G., Faccioli N., Lanza M., Zoppini G., Zanolin E., Schena F., Bonora E., Moghetti P. (2013). Both resistance training and aerobic training reduce hepatic fat content in type 2 diabetic subjects with nonalcoholic fatty liver disease (the RAED2 randomized trial). Hepatology.

[202] Lee S.J., Bacha F., Hannon T., Kuk J.L., Boesch C., Arslanian S. (2012). Effects of aerobic versus resistance exercise without caloric restriction on abdominal fat, intrahepatic lipid, and insulin sensitivity in obese adolescent boys a randomized, controlled trial. Diabetes.

[203] Thyfault J.P., Scott Rector R. (2020). Exercise combats hepatic steatosis: Potential mechanisms and clinical implications. Diabetes.

[204] Hvid T., Lindegaard B., Winding K., Iversen P., Brasso K., Solomon T.P.J., Pedersen B.K., Hojman P. (2016). Effect of a 2-year home-based endurance training intervention on physiological function and PSA doubling time in prostate cancer patients. Cancer Causes Control.

[205] Holloszy J.O. (1967). Biochemical adaptations in muscle. Effects of exercise on mitochondrial oxygen uptake and respiratory enzyme activity in skeletal muscle. J. Biol. Chem..

[206] Granata C., Jamnick N.A., Bishop D.J. (2018). Training-Induced Changes in Mitochondrial Content and Respiratory Function in Human Skeletal Muscle. Sport. Med..

[207] Khadir A., Tiss A., Abubaker J., Abu-Farha M., Al-Khairi I., Cherian P., John J., Kavalakatt S., Warsame S., Al-Madhoun A. (2015). MAP kinase phosphatase DUSP1 is overexpressed in obese humans and modulated by physical exercise. Am. J. Physiol. Metab..

[208] Ruschke K., Fishbein L., Dietrich A., Klöting N., Tönjes A., Oberbach A., Fasshauer M., Jenkner J., Schön M.R., Stumvoll M. (2010). Gene expression of PPARγ and PGC-1α in human omental and subcutaneous adipose tissues is related to insulin resistance markers and mediates beneficial effects of physical training. Eur. J. Endocrinol..

[209] Rönn T., Volkov P., Tornberg Å., Elgzyri T., Hansson O., Eriksson K.-F., Groop L., Ling C. (2014). Extensive changes in the transcriptional profile of human adipose tissue including genes involved in oxidative phosphorylation after a 6-month exercise intervention. Acta Physiol..

[210] Riis S., Christensen B., Nellemann B., Møller A.B., Husted A.S., Pedersen S.B., Schwartz T.W., Jørgensen J.O.L., Jessen N. (2019). Molecular adaptations in human subcutaneous adipose tissue after ten weeks of endurance exercise training in healthy males. J. Appl. Physiol..

[211] Stallknecht B., Vinten J., Ploug T., Galbo H. (1991). Increased activities of mitochondrial enzymes in white adipose tissue in trained rats. Am. J. Physiol. Metab..

[212] Stanford K.I., Middelbeek R.J.W., Townsend K.L., Lee M.-Y., Takahashi H., So K., Hitchcox K.M., Markan K.R., Hellbach K., Hirshman M.F. (2015). A novel role for subcutaneous adipose tissue in exercise-induced improvements in glucose homeostasis. Diabetes.

[213] Lehnig A.C., Stanford K.I. (2018). Exercise-induced adaptations to white and brown adipose tissue. J. Exp. Biol..

[214] Stinkens R., Brouwers B., Jocken J.W., Blaak E.E., Teunissen-Beekman K.F., Hesselink M.K., van Baak M.A., Schrauwen P., Goossens G.H. (2018). Exercise training-induced effects on the abdominal subcutaneous adipose tissue phenotype in humans with obesity. J. Appl. Physiol..

[215] Camera D.M., Anderson M.J., Hawley J.A., Carey A.L. (2010). Short-term endurance training does not alter the oxidative capacity of human subcutaneous adipose tissue. Eur. J. Appl. Physiol..

[216] Fletcher J.A., Meers G.M., Linden M.A., Kearney M.L., Morris E.M., Thyfault J.P., Rector R.S. (2014). Impact of Various Exercise Modalities on Hepatic Mitochondrial Function. Med. Sci. Sport. Exerc..

[217] Laye M.J., Rector R.S., Borengasser S.J., Naples S.P., Uptergrove G.M., Ibdah J.A., Booth F.W., Thyfault J.P. (2009). Cessation of daily wheel running differentially alters fat oxidation capacity in liver, muscle, and adipose tissue. J. Appl. Physiol..

[218] Hock M.B., Kralli A. (2009). Transcriptional Control of Mitochondrial Biogenesis and Function. Annu. Rev. Physiol..

[219] Lin J., Handschin C., Spiegelman B.M. (2005). Metabolic control through the PGC-1 family of transcription coactivators. Cell Metab..

[220] Patti M.E., Corvera S. (2010). The role of mitochondria in the pathogenesis of type 2 diabetes. Endocr. Rev..

[221] Ballarò R., Beltrà M., De Lucia S., Pin F., Ranjbar K., Hulmi J.J., Costelli P., Penna F. (2019). Moderate exercise in mice improves cancer plus chemotherapy-induced muscle wasting and mitochondrial alterations. FASEB J..

[222] RANJBAR K., BALLARÒ R., BOVER Q., PIN F., BELTRÀ M., PENNA F., COSTELLI P. (2019). Combined Exercise Training Positively Affects Muscle Wasting in Tumor-Bearing Mice. Med. Sci. Sport. Exerc..

[223] Pin F., Busquets S., Toledo M., Camperi A., Lopez-Soriano F.J., Costelli P., Argilés J.M., Penna F. (2015). Combination of exercise training and erythropoietin prevents cancer-induced muscle alterations. Oncotarget.

[224] Padrão A.I., Figueira A.C.C., Faustino-Rocha A.I., Gama A., Loureiro M.M., Neuparth M.J., Moreira-Gonçalves D., Vitorino R., Amado F., Santos L.L. (2017). Long-term exercise training prevents mammary tumorigenesis-induced muscle wasting in rats through the regulation of TWEAK signalling. Acta Physiol..

[225] Mijwel S., Cardinale D.A., Norrbom J., Chapman M., Ivarsson N., Wengström Y., Sundberg C.J., Rundqvist H. (2018). Exercise training during chemotherapy preserves skeletal muscle fiber area, capillarization, and mitochondrial content in patients with breast cancer. FASEB J..

[226] Martins R.A., Neves A.P., Coelho-Silva M.J., Veríssimo M.T., Teixeira A.M. (2010). The effect of aerobic versus strength-based training on high-sensitivity C-reactive protein in older adults. Eur. J. Appl. Physiol..

[227] Balducci S., Zanuso S., Cardelli P., Salerno G., Fallucca S., Nicolucci A., Pugliese G. (2012). Supervised exercise training counterbalances the adverse effects of insulin therapy in overweight/obese subjects with type 2 diabetes. Diabetes Care.

[228] Gomez-Merino D., Drogou C., Guezennec C.Y., Chennaoui M. (2007). Effects of chronic exercise on cytokine production in white adipose tissue and skeletal muscle of rats. Cytokine.

[229] Vieira V.J., Valentine R.J., Wilund K.R., Antao N., Baynard T., Woods J.A. (2009). Effects of exercise and low-fat diet on adipose tissue inflammation and metabolic complications in obese mice. Am. J. Physiol. Metab..

[230] Vieira V.J., Valentine R.J., Wilund K.R., Woods J.A. (2009). Effects of diet and exercise on metabolic disturbances in high-fat diet-fed mice. Cytokine.

[231] Bruun J.M., Helge J.W., Richelsen B., Stallknecht B. (2006). Diet and exercise reduce low-grade inflammation and macrophage infiltration in adipose tissue but not in skeletal muscle in severely obese subjects. Am. J. Physiol. Endocrinol. Metab..

[232] Fabre O., Ingerslev L.R., Garde C., Donkin I., Simar D., Barrès R. (2018). Exercise training alters the genomic response to acute exercise in human adipose tissue. Epigenomics.

[233] Pedersen B.K. (2011). Muscles and their myokines. J. Exp. Biol..

[234] Pedersen L., Idorn M., Olofsson G.H., Lauenborg B., Nookaew I., Hansen R.H., Johannesen H.H., Becker J.C., Pedersen K.S., Dethlefsen C. (2016). Voluntary running suppresses tumor growth through epinephrine- and IL-6-dependent NK cell mobilization and redistribution. Cell Metab..

[235] Khosravi N., Stoner L., Farajivafa V., Hanson E.D. (2019). Exercise training, circulating cytokine levels and immune function in cancer survivors: A meta-analysis. Brain. Behav. Immun..

[236] Hiensch A.E., Mijwel S., Bargiela D., Wengström Y., May A.M., Rundqvist H. (2020). Inflammation Mediates Exercise Effects on Fatigue in Patients with Breast Cancer. Med. Sci. Sport. Exerc..

[237] Brown J.C., Zhang S., Ligibel J.A., Irwin M.L., Jones L.W., Campbell N., Pollak M.N., Sorrentino A., Cartmel B., Harrigan M. (2020). Effect of Exercise or Metformin on Biomarkers of Inflammation in Breast and Colorectal Cancer: A Randomized Trial. Cancer Prev. Res..

[238] Jee H., Chang J.-E., Yang E.J. (2016). Positive Prehabilitative Effect of Intense Treadmill Exercise for Ameliorating Cancer Cachexia Symptoms in a Mouse Model. J. Cancer.

[239] Joanisse S., Lim C., McKendry J., Mcleod J.C., Stokes T., Phillips S.M. (2020). Recent advances in understanding resistance exercise training-induced skeletal muscle hypertrophy in humans. F1000Research.

[240] Sigal R.J., Kenny G.P., Boulé N.G., Wells G.A., Prud’homme D., Fortier M., Reid R.D., Tulloch H., Coyle D., Phillips P. (2007). Effects of Aerobic Training, Resistance Training, or Both on Glycemic Control in Type 2 Diabetes. Ann. Intern. Med..

[241] Church T.S., Blair S.N., Cocreham S., Johannsen N., Johnson W., Kramer K., Mikus C.R., Myers V., Nauta M., Rodarte R.Q. (2010). Effects of Aerobic and Resistance Training on Hemoglobin A 1c Levels in Patients With Type 2 Diabetes. JAMA.

[242] Schiaffino S., Reggiani C., Akimoto T., Blaauw B. (2020). Molecular Mechanisms of Skeletal Muscle Hypertrophy. J. Neuromuscul. Dis..

[243] Saxton R.A., Sabatini D.M. (2017). mTOR Signaling in Growth, Metabolism, and Disease. Cell.

[244] Tipton K.D., Hamilton D.L., Gallagher I.J. (2018). Assessing the Role of Muscle Protein Breakdown in Response to Nutrition and Exercise in Humans. Sport. Med..

[245] Solheim T.S., Laird B.J.A., Balstad T.R., Stene G.B., Bye A., Johns N., Pettersen C.H., Fallon M., Fayers P., Fearon K. (2017). A randomized phase II feasibility trial of a multimodal intervention for the management of cachexia in lung and pancreatic cancer. J. Cachexia Sarcopenia Muscle.

[246] Lønbro S., Dalgas U., Primdahl H., Johansen J., Nielsen J.L., Aagaard P., Hermann A.P., Overgaard J., Overgaard K. (2013). Progressive resistance training rebuilds lean body mass in head and neck cancer patients after radiotherapy – Results from the randomized DAHANCA 25B trial. Radiother. Oncol..

[247] Schmitt J., Lindner N., Reuss-Borst M., Holmberg H.-C., Sperlich B. (2016). A 3-week multimodal intervention involving high-intensity interval training in female cancer survivors: A randomized controlled trial. Physiol. Rep..

[248] Mijwel S., Backman M., Bolam K.A., Olofsson E., Norrbom J., Bergh J., Sundberg C.J., Wengström Y., Rundqvist H. (2018). Highly favorable physiological responses to concurrent resistance and high-intensity interval training during chemotherapy: The OptiTrain breast cancer trial. Breast Cancer Res. Treat..

[249] Adamsen L., Quist M., Andersen C., Møller T., Herrstedt J., Kronborg D., Baadsgaard M.T., Vistisen K., Midtgaard J., Christiansen B. (2009). Effect of a multimodal high intensity exercise intervention in cancer patients undergoing chemotherapy: Randomised controlled trial. BMJ.

[250] Lønbro S., Farup J., Bentsen S., Voss T., Rittig N., Wang J., Ørskov M., Højris I., Mikkelsen U.R. (2017). Lean body mass, muscle fibre size and muscle function in cancer patients during chemotherapy and 10 weeks exercise. JCSM Clin. Reports.

[251] Stene G.B., Helbostad J.L., Balstad T.R., Riphagen I.I., Kaasa S., Oldervoll L.M. (2013). Effect of physical exercise on muscle mass and strength in cancer patients during treatment—A systematic review. Crit. Rev. Oncol. Hematol..

[252] Lee J. (2021). The effects of resistance training on muscular strength and hypertrophy in elderly cancer patients: A systematic review and meta-analysis. J. Sport Heal. Sci..

[253] Tanaka M., Sugimoto K., Fujimoto T., Xie K., Takahashi T., Akasaka H., Kurinami H., Yasunobe Y., Matsumoto T., Fujino H. (2019). Preventive effects of low-intensity exercise on cancer cachexia–induced muscle atrophy. FASEB J..

[254] Salomão E.M., Toneto A.T., Silva G.O., Gomes-Marcondes M.C.C. (2010). Physical Exercise and a Leucine-Rich Diet Modulate the Muscle Protein Metabolism in Walker Tumor-Bearing Rats. Nutr. Cancer.

[255] Khamoui A.V., Park B.-S., Kim D.-H., Yeh M.-C., Oh S.-L., Elam M.L., Jo E., Arjmandi B.H., Salazar G., Grant S.C. (2016). Aerobic and resistance training dependent skeletal muscle plasticity in the colon-26 murine model of cancer cachexia. Metabolism.

[256] Tanaka M., Sugimoto K., Fujimoto T., Xie K., Takahashi T., Akasaka H., Yasunobe Y., Takeya Y., Yamamoto K., Hirabayashi T. (2020). Differential effects of pre-exercise on cancer cachexia-induced muscle atrophy in fast- and slow-twitch muscles. FASEB J..

[257] Hardee J.P., Fix D.K., Koh H.-J., Wang X., Goldsmith E.C., Carson J.A. (2020). Repeated eccentric contractions positively regulate muscle oxidative metabolism and protein synthesis during cancer cachexia in mice. J. Appl. Physiol..

[258] Staples A.W., Burd N.A., West D.W.D., Currie K.D., Atherton P.J., Moore D.R., Rennie M., Macdonald M.J., Baker S.K., Phillips S.M. (2011). Carbohydrate Does Not Augment Exercise-Induced Protein Accretion versus Protein Alone. Med. Sci. Sport. Exerc..

[259] Pennings B., Koopman R., Beelen M., Senden J.M., Saris W.H., van Loon L.J. (2011). Exercising before protein intake allows for greater use of dietary protein–derived amino acids for de novo muscle protein synthesis in both young and elderly men. Am. J. Clin. Nutr..

[260] Turner R.R., Steed L., Quirk H., Greasley R.U., Saxton J.M., Taylor S.J., Rosario D.J., Thaha M.A., Bourke L. (2018). Interventions for promoting habitual exercise in people living with and beyond cancer. Cochrane database Syst. Rev..

[261] Schmitz K.H., Courneya K.S., Matthews C., Demark-Wahnefried W., GALVÃO D.A., Pinto B.M., IRWIN M.L., WOLIN K.Y., SEGAL R.J., LUCIA A. (2010). American College of Sports Medicine Roundtable on Exercise Guidelines for Cancer Survivors. Med. Sci. Sport. Exerc..

